# Galectin-3 acts as an angiogenic switch to induce tumor angiogenesis via Jagged-1/Notch activation

**DOI:** 10.18632/oncotarget.17718

**Published:** 2017-05-09

**Authors:** Sofia Nascimento dos Santos, Helen Sheldon, Jonathas Xavier Pereira, Christopher Paluch, Esther M Bridges, Márcia Curry El-Cheikh, Adrian L Harris, Emerson Soares Bernardes

**Affiliations:** ^1^ Radiopharmacy Department, Nuclear Energy Research Institute, São Paulo, Brazil; ^2^ Department of Medical Oncology, Molecular Oncology Laboratories, Weatherall Institute of Molecular Medicine, University of Oxford, Oxford, UK; ^3^ Department of Pathology, Faculty of Medicine, Federal University of Rio de Janeiro, Rio de Janeiro, Brazil; ^4^ T-cell Biology Group, Human Immunology Unit, Weatherall Institute of Molecular Medicine, University of Oxford, Oxford, UK; ^5^ Institute for Biomedical Sciences, Federal University of Rio de Janeiro, Rio de Janeiro, Brazil

**Keywords:** angiogenesis, cancer, galectin-3, Jagged-1, Notch

## Abstract

Angiogenesis is a coordinated process tightly regulated by the balance between Delta-like-4 (DLL4) and Jagged-1 (JAG1) in endothelial cells. Here we show that galectin-3 (gal-3), a glycan-binding protein secreted by cancer cells under hypoxic conditions, triggers sprouting angiogenesis, assisted by hypoxic changes in the glycosylation status of endothelial cells that enhance binding to gal-3. Galectin-3′s proangiogenic functions were found to be predominantly dependent on the Notch ligand JAG1. Differential direct binding to JAG1 was shown by surface plasmon resonance assay. Upon binding to Notch ligands, gal-3 preferentially increased JAG1 protein half-life over DLL4 and preferentially activated JAG1/Notch-1 signaling in endothelial cells. JAG1 overexpression in Lewis lung carcinoma cells accelerated tumor growth *in vivo*, but this effect was prevented in Lgals3^−/−^ mice. Our findings establish gal-3 as a molecular regulator of the JAG1/Notch-1 signaling pathway and have direct implications for the development of strategies aimed at controlling tumor angiogenesis.

## INTRODUCTION

In tumors, the angiogenic switch occurs when a tumor expands beyond the capacity of its blood supply. An insufficient supply of nutrients and oxygen triggers tumor cells to release hypoxia-dependent proangiogenic signals, such as vascular endothelial growth factor (VEGF) [1], which stimulate quiescent endothelial cells (ECs) to become migratory and invasive. The current, widely accepted, angiogenic model describes that VEGF/VEGFR2 signaling upregulates DLL4 ligand on responsive endothelial tip cells, which signals to the Notch receptor on adjacent endothelial cells (ECs) directing them towards a stalk cell phenotype and repressing VEGFR2 expression, which in turn prevents excess sprout formation [2]. Indeed DLL4/Notch inhibition has been shown to reduce tumor growth by producing an excess proliferation of immature vessels and so deficient vascular perfusion [3]. Unlike DLL4, JAG1 expression in ECs has been shown to have proangiogenic functions; to antagonize DLL4/Notch signaling but also to promote vascular maturation and so to aid tumor growth [4–6]. Since DLL4 and JAG1 have opposing roles in angiogenesis, the identification of upstream signals controlling the expression of one or the other ligand might modulate angiogenesis positively or negatively and have a direct impact on the development of strategies aimed at controlling tumor angiogenesis.

Recently, much attention has been given to galectins (glycan-binding proteins) and their role in tumor angiogenesis [7–9]. Galectin-3 (gal-3), is unique among galectins for having an N-terminal nonlectin domain, in addition to the usual C-terminal carbohydrate recognition domain (CRD). The N-terminal domain allows oligomerization upon recognition of ligands by the CRD thereby promoting cross-linking of ligands on the cell surface [10]. Galectin-3 has been reported as a proangiogenic molecule that is induced during hypoxia [11, 12] and increases the chemotaxis and differentiation of human umbilical vein endothelial cells (HUVECs) by enhancing VEGFR2 signaling activation [13–15]. Disruption of gal-3 in the tumor stroma reduces macrophage induced-angiogenesis dependent on VEGF and TGF-β signaling [16].

Lately, a role for gal-3 in enhancing Notch signaling as been demonstrated. Indeed, gal-3 silencing or overexpression, reduced or increased cleaved Notch-1 intracellular domain (NICD1), respectively, and the expression of the Notch target genes Hes1 and Hey1 in ovarian cancer cells [17]. Moreover, gal-3 CRD was found to interact with NICD1 and its overexpression increased NICD1 nuclear translocation [17]. Soluble gal-3, was also found to regulate bone remodeling through sugar-dependent Notch-1 binding and Notch signaling activation [18]. However, in the context of the immune response, evidence suggests a role for gal-3 in dampening Notch signalling activation. T regulatory and T effector cells from gal-3 knockout mice, had higher expression of Notch-1 and the Notch target *Hes-1* [19]. Lack of gal-3 increased JAG1/Notch activation in bone marrow-derived dendritic cells and promoted dysregulation of T helper cell polarization [20].

Here, we investigated a new mechanism by which gal-3 affects tumor angiogenesis by altering the balance of JAG1 and DLL4 expression and function in ECs. Although the importance of gal-3 in angiogenesis is already widely appreciated, we now point to a novel regulatory mechanism by which tumor-secreted gal-3 increases tip cell formation and sprouting angiogenesis because of its ability to upregulate JAG1/Notch-1 signaling in endothelial cells. This study opens new perspectives for targeting tumor angiogenesis.

## RESULTS

### Galectin-3 binding to endothelial cells is increased under hypoxic conditions

**Figure 1:**
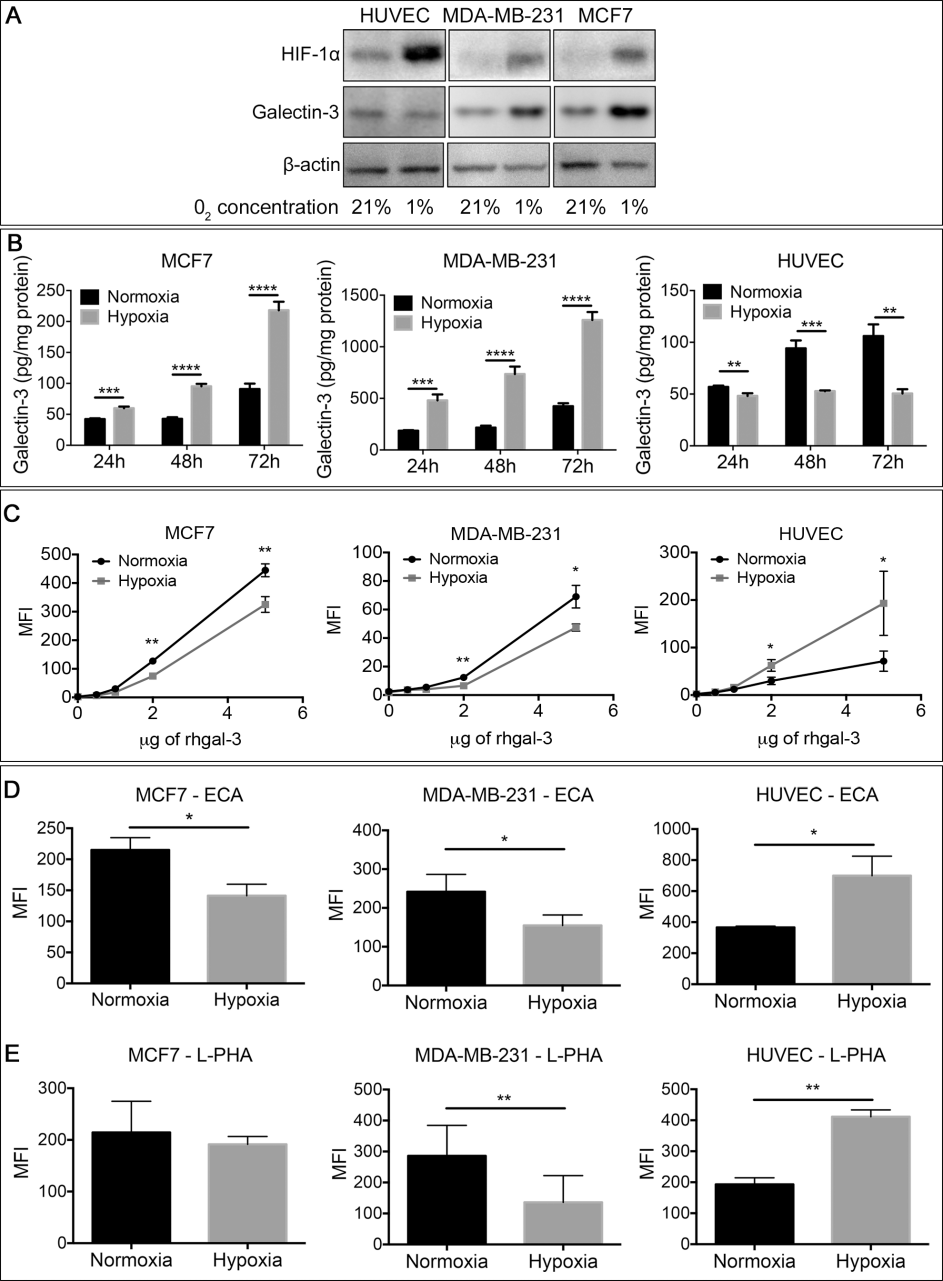
Tumor-secreted galectin-3 under hypoxic conditions increases it's binding to endothelial cells (**A**) Endothelial cells (HUVECs) and the human breast cancer cells MDA-MB-231 and MCF7 were grown under normoxic (21% O2) or hypoxic (1% O2) conditions for 48 hrs. After this period, the total protein was isolated and the protein levels of HIF-1α and galectin-3 were assessed by Western blot. β-actin was used as a loading control. (**B**) MCF7, MDA-MB-231 and HUVECs cells were cultured in a six well plate under normoxic or hypoxic conditions. After 48 hrs, the conditioned medium of cells was collected and gal-3 from the medium was quantified by an ELISA assay. Data are presented as pg/mg of total protein. (**C**) MCF7, MDA-MB-231 and HUVEC were cultured under normoxic or hypoxic conditions. After 48 hrs, cells were collected and incubated with DyLigth488 labeled-rhgal-3. Gal-3 binding was evaluated by flow cytometry and data are presented as the mean fluorescence intensity. (**D**) and (**E**) Flow cytometry of MCF7, MDA-MB-231 and HUVECs detected with the biotinylated lectins (D) ECA and (E) L-PHA or with Cy5-conjugated streptavidin alone after culture under normoxic or hypoxic conditions for 48 hrs. Data are presented as the mean fluorescence intensity. Data are (A) representative of three independent experiments or (B–E) the mean (S.D.), *n* = 3. *p < 0.05, **p < 0.01, ***p < 0.001, ****p < 0.0001 by one-way ANOVA and two-tailed unpaired Student's *t-test*

Hypoxia is the primary physiological trigger of tumor angiogenesis [21] by stimulating the production of several proangiogenic factors [22] including gal-3 [11, 23] by tumor cells. Accordingly, under hypoxic conditions, MCF7 and MDA-MB-231 human breast cancer cells increased the protein (Figure 1A), mRNA expression (Supplementary Figure 1A) and secreted levels (Figure 1B) of gal-3 in comparison to normoxic conditions. In contrast, gal-3 was reduced in human umbilical vein endothelial cells (HUVECs) under hypoxia. We analyzed gal-3 binding to breast cancer cells and HUVECs under hypoxic conditions and found a reduction of DyLight488-labeled-rhgal-3 binding to the cell surface of hypoxic MCF7 and MDA-MB-231 cells in comparison to normoxic cells (Figure 1C). In contrast, we found higher binding of DyLight488-labeled-rhgal-3 to HUVECs cultured under hypoxic conditions (Figure 1C).

We then used a panel of lectins (Supplementary Figure 1B) to evaluate the changes in cell surface glycans on hypoxic HUVECs that may be contributing to increased gal-3 binding. We found that hypoxic HUVECs showed increased reactivity to ECA lectin (*Erythrina Cristagalli*) (Figure 1D), which recognizes unsialylated terminal galactosyl (β-1,4) N-acetylglucosamine, and to L-phythemagglutinin (L-PHA) (Figure 1E), which recognizes tri- and tetraantennary complex-type *N*-glycans. In contrast, we observed a decrease in ECA and L-PHA reactivity (Figure 1D and 1E) in hypoxic MCF7 and MDA-MB-231 cells in comparison to normoxic conditions. Thus, hypoxia favors the formation of a different glycophenotype that increases the binding of gal-3 to endothelial cells versus cancer cells.

### Galectin-3 secretion by cancer cells increases sprouting angiogenesis via JAG1 ligand

We next sought to investigate whether tumor-secreted gal-3 primes ECs for angiogenic sprouting. We found that exogenously added recombinant human gal-3 (rhgal-3) played an important role in HUVEC proliferation (Supplementary Figure 2A) and increased sprouting angiogenesis by HUVECs (Supplementary Figure 2B). These effects were inhibited by lactose and were thus dependent on the gal-3 carbohydrate-binding domain.

**Figure 2:**
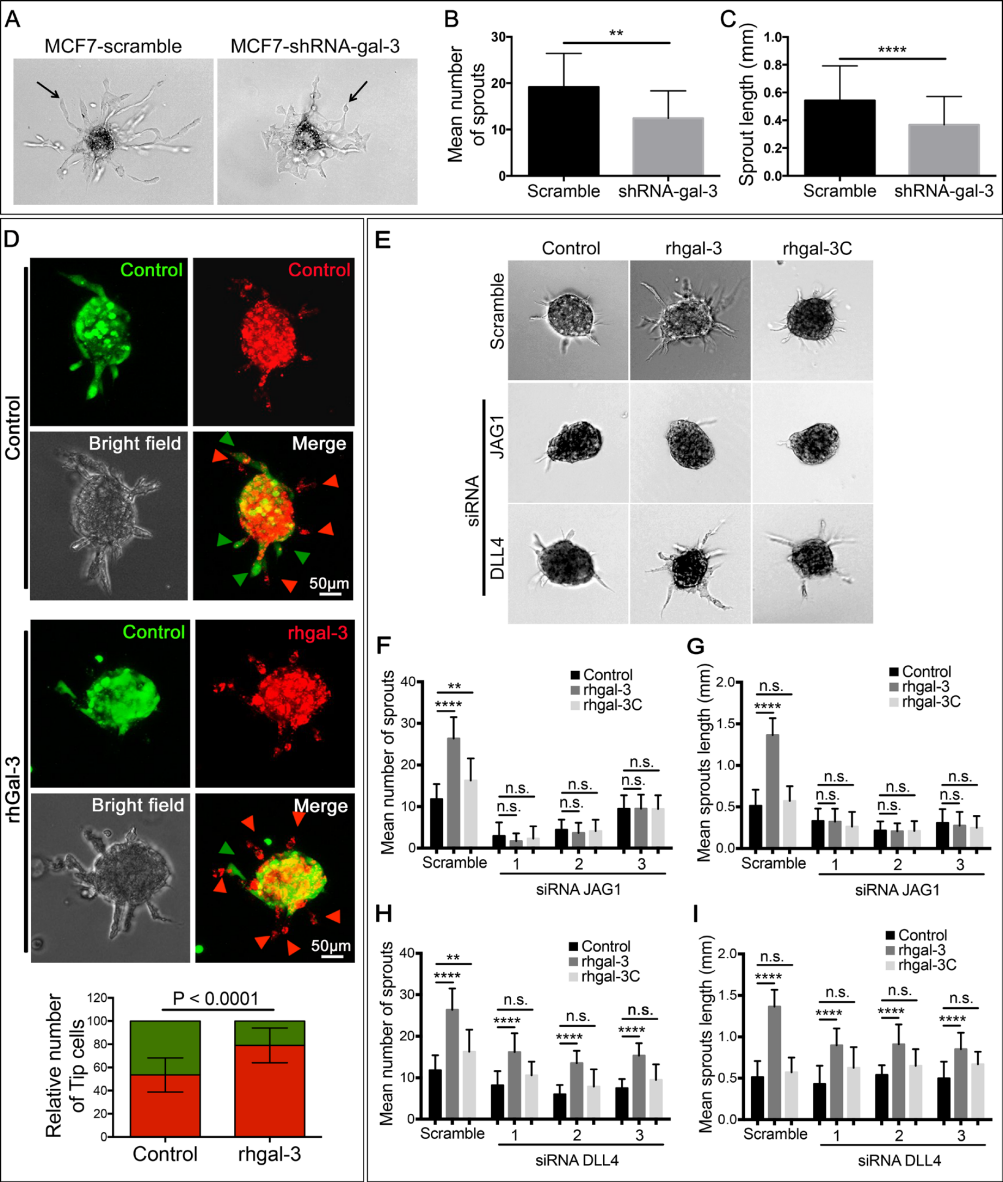
Galectin-3 secretion by cancer cells increases sprouting angiogenesis via JAG1 ligand (**A–C**) HUVECs spheroids embedded in a fibrinogen gel were cultured for 24 hrs with MCF7-scramble or shRNA-gal-3 cells seeded on the top of the gel. After this period, spheroids were (A) photographed and (B) the average number and (C) sprouts length were quantified. (**D**) *In vitro* cell fate assay. Prior to spheroids formation, HUVECs were labeled with cell tracker green or cell tracker red and then mixed in a 1:1 ratio. Alternatively, red-labeled HUVECs were incubated for 15 min with rhgal-3 (37 nM) prior to spheroid formation. Spheroids were then embedded in a fibrinogen gel and cultured for 24 hrs. Arrowheads indicate the tip cells position and graph shows the percentage of green or red-labeled tip cells found per spheroid. (**E–I**) HUVECs previously transfected with JAG1 or DLL4 siRNA were grown into spheroids overnight in the presence/absence of rhgal-3 or rhgal-3C (37 nM). After this period spheroids were embedded in fibrinogen gel and cultured for additional 24 hrs (E) representative images are shown. (F and H) Mean number of sprouts and (G and I) sprouts length of HUVECs spheroids were measured. Controls are the same for F-I and all conditions were run simultaneously for each replicates Data are (A, D and E) representative images or (B–D and F–I) the mean (S.D.) of three independent experiments, *n* = 20. **p < 0.01, ****p < 0.0001 by 2-way ANOVA or two-tailed paired Student's *t-test*

We subsequently silenced MCF7 breast cancer cells with gal-3 shRNA, and confirmed reduced levels of protein, mRNA and secreted gal-3 in comparison to the scrambled control (Supplementary Figure 2C–2E). We then cultured scrambled and gal-3-silenced cells on the top of HUVEC spheroids. In the presence of gal-3-silenced MCF7 cells, both the number of sprouts (Figure 2A and 2B) and the sprout length (Figure 2A and 2C) were significantly lower than when grown with MCF7-scramble cells.

To investigate the role of gal-3 in the induction of a tip cell phenotype we mixed red or green-pre-labeled HUVECs in a 1:1 ratio for spheroid formation and found that approximately 50% of ECs at the growing tip position were red or green. When red-labeled HUVECs were treated with rhgal-3 prior to spheroid formation, the probability of detecting a red-labeled cell at the tip growing position increased significantly, from 50 ± 13.75% to 79 ± 14.76% (*p <* 0,0001) (Figure 2D).

Since the balance between JAG1 and DLL4 coordinates the process of tip cell selection, we further silenced JAG1 or DLL4 in HUVECs with 3 different siRNAs (Supplementary Figure 2F) and found that the ability of rhgal-3 to increase the number (Figure 2E, 2F and 2H) and length (Figure 2E, 2G and 2I) of spheroid sprout was completely impaired in JAG1-silenced HUVECs but not in DLL4-silenced HUVECs or the scramble control. In contrast to rhgal-3, the N-terminally truncated recombinant human gal-3 (rhgal-3C), which lacks the ability to oligomerize, had a much smaller increase in number of sprouts, and no increase in their length (Figure 2F–2I), and similar effects to the negative controls in the JAG1 and DLL4-silenced HUVECs. Note that there are no significant effects in the number of sprouts in DLL4-silenced HUVECs, suggesting the effect on the siRNA controls are of minor importance. These results show oligomerization is needed for the interaction with JAG1 and DLL4.

It was previously shown that JAG1 is enriched in tip cells [24] and that loss of JAG1 in ECs significantly decreases the number of tip cells [4]. Interestingly, we observed that following rhgal-3 treatment, JAG1 expression increased in HUVECs cultured in a monolayer (Supplementary Figure 2G) and was enriched in the tip cells of HUVECs spheroids (Supplementary Figure 2H). No differences in DLL4 expression were found in either condition. Altogether, these findings show that gal-3 proangiogenic functions are at least in part JAG1-dependent.

### Galectin-3 binds and selectively increases JAG1 versus DLL4 half-life

**Figure 3:**
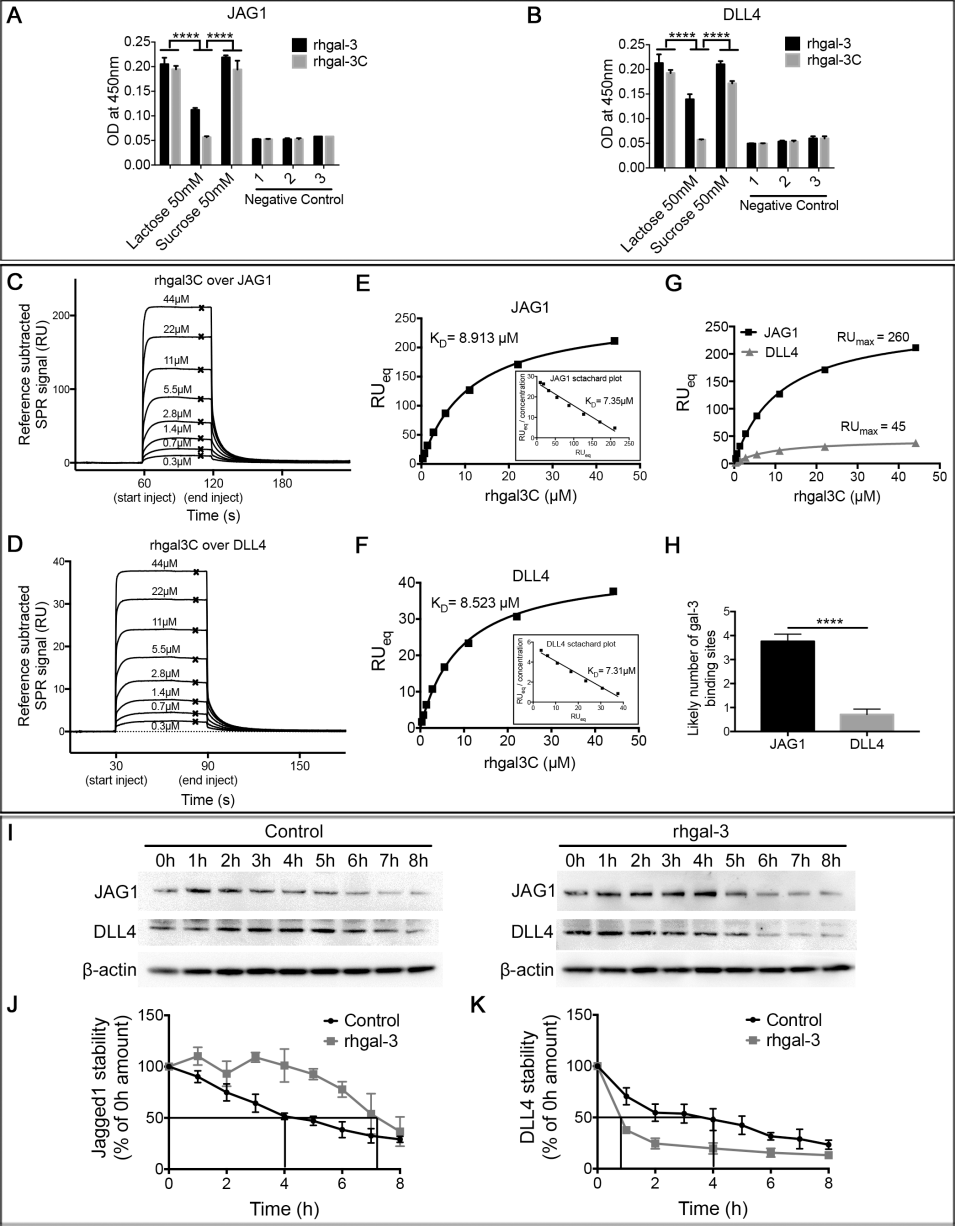
Galectin-3 binds and increases JAG1 half-life over DLL4 (**A**) and (**B**) ELISA detection of rhgal-3 or rhgal-3C in a 96 well plate previously coated with the recombinant human (A) JAG1 or (B) DLL4 proteins in the presence or absence of lactose (50 mM) or sucrose (50 mM). (**C–F**). (C and D) Reference subtracted SPR signal for serial dilutions of rhgal-3C flowed over immobilized (C) JAG1 or (D) DLL4, crosses show binding level at equilibrium used to plot Langmuir binding curve. (E and F) Langmuir binding isotherm and Scatchard plot (inset) of rhgal-3C (0.345 μM–44.1 μM) binding to immobilized (E) JAG1 or (F) DLL4. (**G**) RU_MAX_ of rhgal-3C binding to JAG1 and DLL4 immobilized at similar molar amounts. (**H**) Estimation of the number of exposed gal-3 binding sites per immobilized molecule of JAG1 or DLL4. (**I**) HUVECs previously treated with 37 nM of rhgal-3 for 15 min were cultured for 8 hrs in the presence of 0.1 mM of cycloheximide to block protein synthesis. Immunoblot of JAG1 and DLL4 in each time point (0–8 h) are presented and β-actin was used as a loading control. (**J**) and (**K**) Signal based on densitometry analysis of blots for (J) JAG1 and (K) DLL4 from 3 independent experiments. Quantification of Western blot bands showed a prolonged half-life of JAG1 protein (240 min to 400 min) and an increased degradation rate of DLL4 (150 min to 50 min). The effect on JAG1 and DLL4 stability was statistically significant *p <* 0.01. Data are the mean (S.D.), *n* = 3 (A, B, J and K) or are representative (C–G and I) of four independent experiments. ***p < 0.001, ****p < 0.0001 by 2-way ANOVA or two-tailed paired Student's *t-test*

Since Notch ligands are cell surface glycoproteins that display terminal β-galactose [25], which might be recognized by gal-3, we analyzed the binding of gal-3 to JAG1 and DLL4 ligands. We found that both rhgal-3 and rhgal-3C were able to bind the recombinant human JAG1 (Figure 3A) and DLL4 (Figure 3B) previously immobilized on an ELISA plate. In the presence of β-lactose (a competitive inhibitor of the gal-3 CRD), both rhgal-3 and rhgal-3C binding to Notch ligands were inhibited. Sucrose, a non-competitive saccharide, was used as a control and had no effect on rhgal-3 and rhgal-3C binding.

The binding of gal-3 to JAG1 and DLL4 ligands, were confirmed in a surface plasmon resonance (SPR) assay. JAG1 or DLL4 were covalently immobilized via amine coupling onto the chip surface, with rhgal-3 or rhgal-3C flowed over as analyte. Our data showed that rhgal-3 bound both JAG1 and DLL4 (Supplementary Figure 3A and 3B) and the binding was blocked in the presence of lactose, but not sucrose (Supplementary Figure 3C and 3D). Rhgal-3 showed positive cooperativity of binding, presumably due to oligomerization via its N-terminal domain. Therefore, rhgal-3C (which lacks the N-terminal domain) was used to ascertain the affinity of the monomeric interactions between the gal-3 CRD and JAG1 (Figure 3C and 3E) or DLL4 (Figure 3D and 3F). The dissociation constant (K_D_) was determined by non-linear fitting of the SPR signal at equilibrium for increasing concentrations of analyte, using the Langmuir binding isotherm (K_D_ 8.913 ± 0.485 μM for JAG1 and 8.523 ± 0.335 μM for DLL4). When similar molar amounts of JAG1 and DLL4 were immobilized on the chip, we observed a significantly greater maximum binding of rhgal-3C to JAG1 in comparison to DLL4 (Figure 3G). Using the relative molecular weights of the different proteins we calculated the molar ratio of maximum bound analyte to immobilized ligand and found that JAG1 had an average of 3,7588 ± 0,302 exposed gal-3 binding sites per immobilized molecule in comparison with DLL4 that only presents an average of 0,7088 ± 0,226 gal-3 binding sites (Figure 3H), approximating the 5 fold difference in directly measured binding.

Using NetNGlyc 1.0 [26] and NetOGlyc 4.0 [27] softwares for bioinformatics prediction of N- and O-glycans site occupancy, respectively, we found 8 predicted individual sites likely to carry N-glycans and 57 sites likely to carry O-GalNAc modifications in JAG1 protein. On the other hand, DLL4 was predicted to likely carry 4 N-glycans and 24 O-glycans modifications, which are approximately twice less glycosylation sites than the ones predicted for JAG1.

Since gal-3 affects the cellular distribution and turnover rate of cell surface glycoproteins [14], we next treated HUVECs with cycloheximide and found that rhgal-3 increased the half-life of JAG1 protein from ∼240 min to ∼420 min (Figure 3I and 3J) and decreased DLL4 protein half-life from ∼240 min to ∼50 min (Figure 3I and 3K). To evaluate whether gal-3-increased accumulation of JAG1 on the plasma membrane could be a result of reduced internalization and degradation of the ligand, we purified HUVEC cell surface and intracellular proteins separately following rhgal-3 treatment (Supplementary Figure 3E). We found that treatment with rhgal-3 decreased the intracellular fraction of JAG1, but increased its expression at the cell surface when compared to the control.

Overall these results demonstrate that gal-3 is capable of binding to both JAG1 and DLL4 ligands, but selectively increases JAG1 half-life and its accumulation at the cell surface.

### Galectin-3 increases activation of Notch signaling in endothelial cells

**Figure 4:**
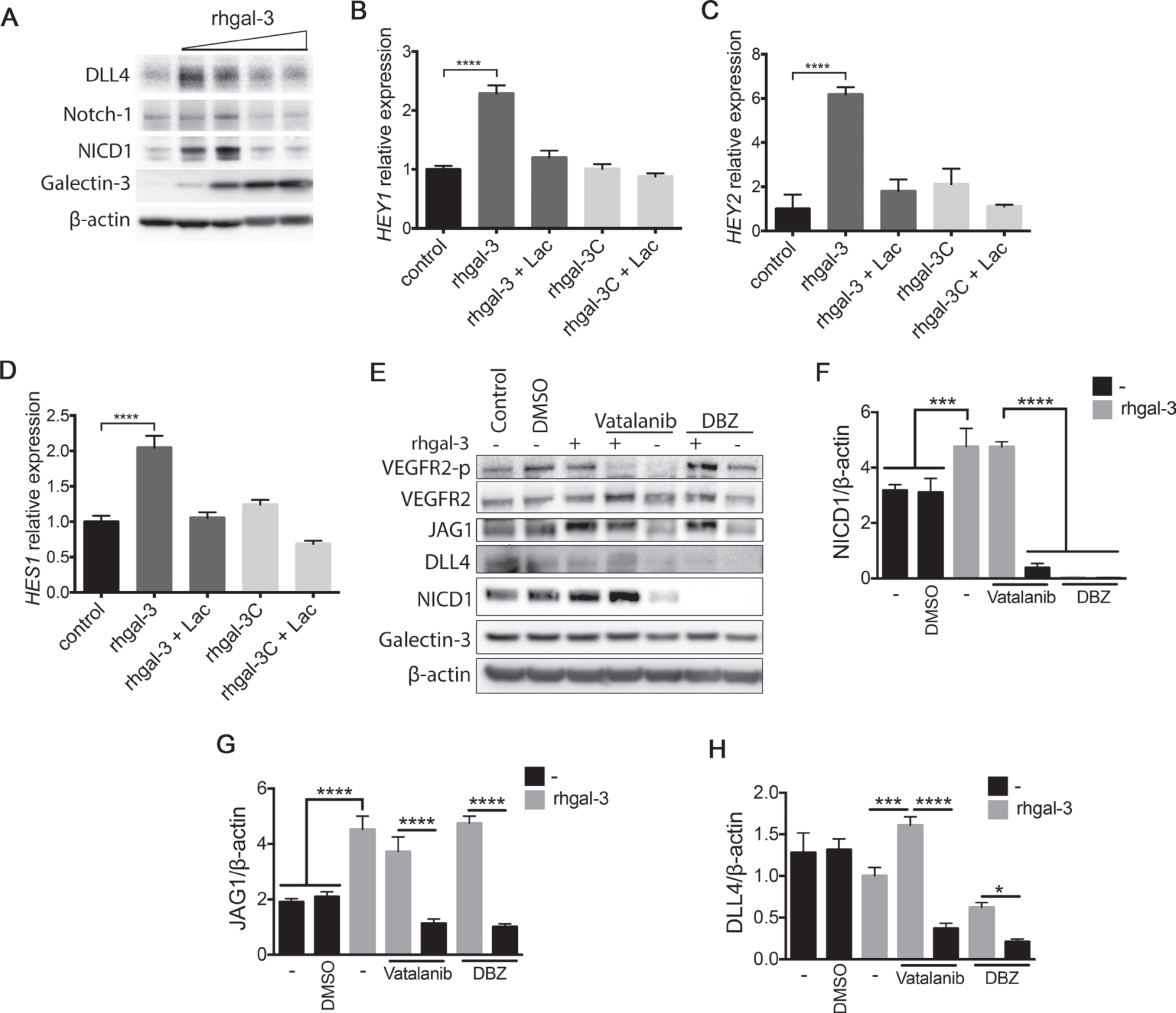
Galectin-3 increases Notch signaling activation in endothelial cells (**A**) HUVECs were treated with 0.037, 0.370, 0.925 or 1.85 μM of rhgal-3 for 6 hrs. After this period, the total protein was isolated and the protein levels of galectin-3, NICD1, Notch-1 and DLL4 were assessed by Western blot. β-actin was used as a loading control. (**B–D**) (B) *HEY1*, (C) *HEY2* and (D) *HES1* mRNA levels of HUVECs cultured for 6 hrs with 37 nM of rhgal-3 or rhgal-3C in the presence or absence of lactose (50 mM). (**E**) HUVECs were cultured in the presence of Vatalanib (100 nM) or DBZ (10 nM) for 1 h and then treated for 15 min with rhgal-3 (37 nM) and cultured for additional 6 hrs. After this period, the total protein was isolated and the protein levels of galectin-3, NICD1, DLL4, JAG1, VEGFR2 and VEGFR2-p were assessed by Western blot. β-actin was used as a loading control. (**F–H**) Average signal of (F) NICD1, (G) JAG1 and (H) DLL4 based on densitometry analysis of blots from 3 independent experiments. Data are the mean (S.D.), *n* = 3 (B–D, F–H) or are representative (A and E) of three independent experiments. *p < 0.05, ***p < 0.001, ****p < 0.0001 by one-way ANOVA

We subsequently investigated whether gal-3 modulated activation of Notch signaling in HUVECs. We found that low doses of rhgal-3 (37 and 370 nM) increased Notch-1 cleavage (NICD1) and up-regulated the protein levels of the Notch target DLL4 (Figure 4A) whereas higher doses of rhgal-3 (0,925 μM and 1,85 μM) had an inhibitory effect on Notch signaling. By measuring the expression of downstream Notch targets we observed that HUVECs treated with rhgal-3 significantly increased the transcription of *HEY1* (Figure 4B), *HEY2* (Figure 4C) and *HES1* (Figure 4D) in comparison to untreated cells, whereas its truncated form, rhgal-3C, had no effect on Notch signaling activation. The effect of rhgal-3 on Notch activation was inhibited by lactose and thus, dependent on its carbohydrate-binding domain.

Since VEGFR2 increases DLL4 levels and consequently Notch signaling [28, 29] and, gal-3 binds and activates VEGFR2 [14, 15], we cultured HUVECs in the presence of Vatalanib (an inhibitor of VEGF receptor kinase). Rhgal-3 treatment alone increased VEGFR2 phosphorylation, the level of NICD1 (Figure 4E and 4F) and JAG1 (Figure 4E and 4G) in comparison with the control. In the presence of Vatalanib and rhgal-3 we found an upregulation of NICD1 and JAG1 levels but not VEGFR2 phosphorylation in comparison with Vatalanib alone, showing that Notch signaling activation by gal-3 is independent of VEGFR2 kinase activity. Vatalanib alone reduced the VEGF-dependent activation of DLL4 and, consequently, the NICD1 levels in comparison to control (Figure 4E and 4H). The inhibition of γ-secretase with dibenzazepine (DBZ), that blocks the cleavage of Notch into its active signaling form, reduced both NICD1 and DLL4 levels in comparison to the DMSO control (Figure 4E, 4F and 4H). Interestingly, rhgal-3 increased JAG1 protein levels in the presence of DBZ and these levels were not associated with an increase in JAG1 mRNA levels (Supplementary Figure 4A). Furthermore, gal-3 treatment also led to an increase in the molecular weight of JAG1 suggesting possible protein modifications have occurred such as phosphorylation (Figure 4E). Following HUVECs cultured with immobilized JAG1 (Supplementary Figure 4B) or DLL4 (Supplementary Figure 4C), rhgal-3 was also found to activate Notch signaling in a VEGFR2 independent way and to increase protein levels of JAG1 and the Notch target DLL4. Together, our results demonstrate that gal-3 activates Notch signaling in a concentration and carbohydrate-dependent manner. Moreover, gal-3 induced-Notch signaling activation is independent of VEGF/VEGFR2 signaling.

### Tumor-secreted galectin-3 increases JAG1/Notch signaling in endothelial cells

**Figure 5:**
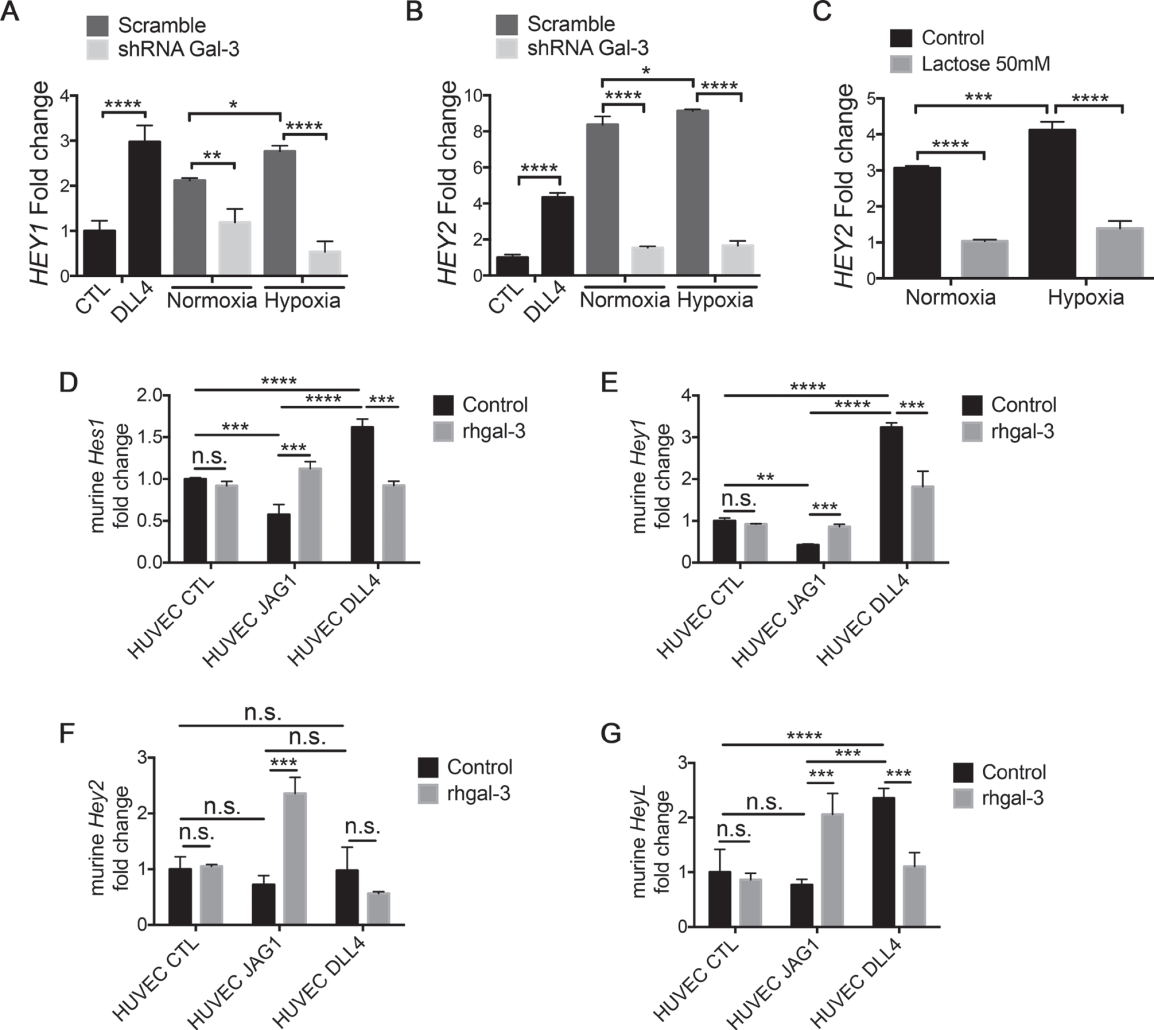
Tumor-secreted galectin-3 increases JAG1/Notch signaling activation in endothelial cells (**A**) and (**B**) HUVECs were cultured for 6 hrs in the presence of the conditioned medium obtained from MCF7-scramble or shRNA-gal-3 cells previously grown in normoxic or hypoxic conditions for 48 hrs. As a positive control for Notch signaling activation, HUVECs were also cultured on BSA- (control) or DLL4-coated plated for 6 hrs. After this period, total RNA was extracted and (A) *HEY1* and (B) *HEY2* mRNA levels were evaluated by Real-Time PCR. Relative quantification was done using the ΔΔCt method normalizing to GAPDH gene expression. (**C**) HUVECs were cultured for 6 hrs in the presence of the conditioned medium obtained from MCF7 cells previously grown in normoxic or hypoxic conditions for 48 hrs ± Lactose (50 mM). After this period, total RNA was extracted and *HEY2* mRNA levels were evaluated by Real-Time PCR. Relative quantification was done using the ΔΔCt method normalizing to GAPDH gene expression. (**D–G**) HUVEC-EV, HUVEC-JAG1 or HUVEC-DLL4 were cultured for 6 hrs in a 1:1 ratio with mouse endothelial cells sEnd-1 in the presence or absence of rhgal-3 (37 nM). After this period, total RNA was extracted and murine (D) *Hes1*, (E) *Hey1* and (F) *Hey2* (G) *HeyL* mRNA levels were evaluated by Real-Time PCR. Relative quantification was done using the ΔΔCt method normalizing to GAPDH gene expression. Data are the mean (S.D.), *n* = 3, ***p < 0.001, by two-tailed unpaired Student's *t-test*

We next cultured HUVECs in the presence of MCF7-scramble or MCF7-shRNA-gal-3 conditioned medium to assess the influence of tumor-secreted gal-3 in Notch signaling activation and whether it has a role in a complex secretome induced by hypoxia. The conditioned medium obtained from MCF7-scrambled cells cultured under normoxic or hypoxic conditions significantly increased the levels of Notch target genes *HEY1* (Figure 5A) and *HEY2* (Figure 5B) in HUVECs, as did immobilized DLL4. However Notch signaling activation remained at control levels when HUVECs were treated with conditioned medium from normoxic or hypoxic gal-3-silenced MCF7 cells (Figure 5A and 5B). *HEY2* was more strongly induced then *HEY1* but both increased further with hypoxic conditioned medium in comparison with normoxia. This suggests that even the basal level of conditioned medium contains a soluble activator of Notch signaling, which is lost by gal-3 knockdown. We further studied whether Notch activation by the conditioned medium from normoxic or hypoxic MCF7 cells was dependent on gal-3 carbohydrate-binding domain and found that tumor-released gal-3 activates the Notch target *HEY2* in a lactose inhibitable manner (Figure 5C).

To evaluate if gal-3 modulates the ligand preference for Notch signaling activation between endothelial cells we then tested the ligand induced Notch signaling by JAG1- or DLL4-expressing cells as opposed to tethered proteins, and co-cultured (1:1) JAG1- or DLL4-overexpressing HUVECs with murine endothelial cells sEnd-1 (Supplementary Figure 5A–5C). The ligands JAG1 and DLL4 differentially modified Notch signaling in the murine responder cells. HUVEC-DLL4 increased the mRNA levels of *Hes1* (Figure 5D), *Hey1* (Figure 5E) and *HeyL* (Figure 5G) while HUVEC-JAG1 had no effect or decreased *Hes1*, *Hey1* and *HeyL*. However, addition of rhgal-3 markedly changed these responses in favor of activating JAG1 and inhibiting DLL4 signaling. Accordingly, rhgal-3 increased murine *Hes1* (Figure 5D), *Hey1* (Figure 5E), *Hey2* (Figure 5F) and *HeyL* (Figure 5G) mRNA levels in response to JAG1-overexpressing HUVECs but decreased signaling when co-cultured with DLL4-overexpressing HUVECs (Figure 5D–5G) showing that gal-3 switches the direction of action of Notch activation by JAG1 and DLL4.

### The growth of JAG1 overexpressing tumor cells is impaired in galectin-3 knock out mice

**Figure 6:**
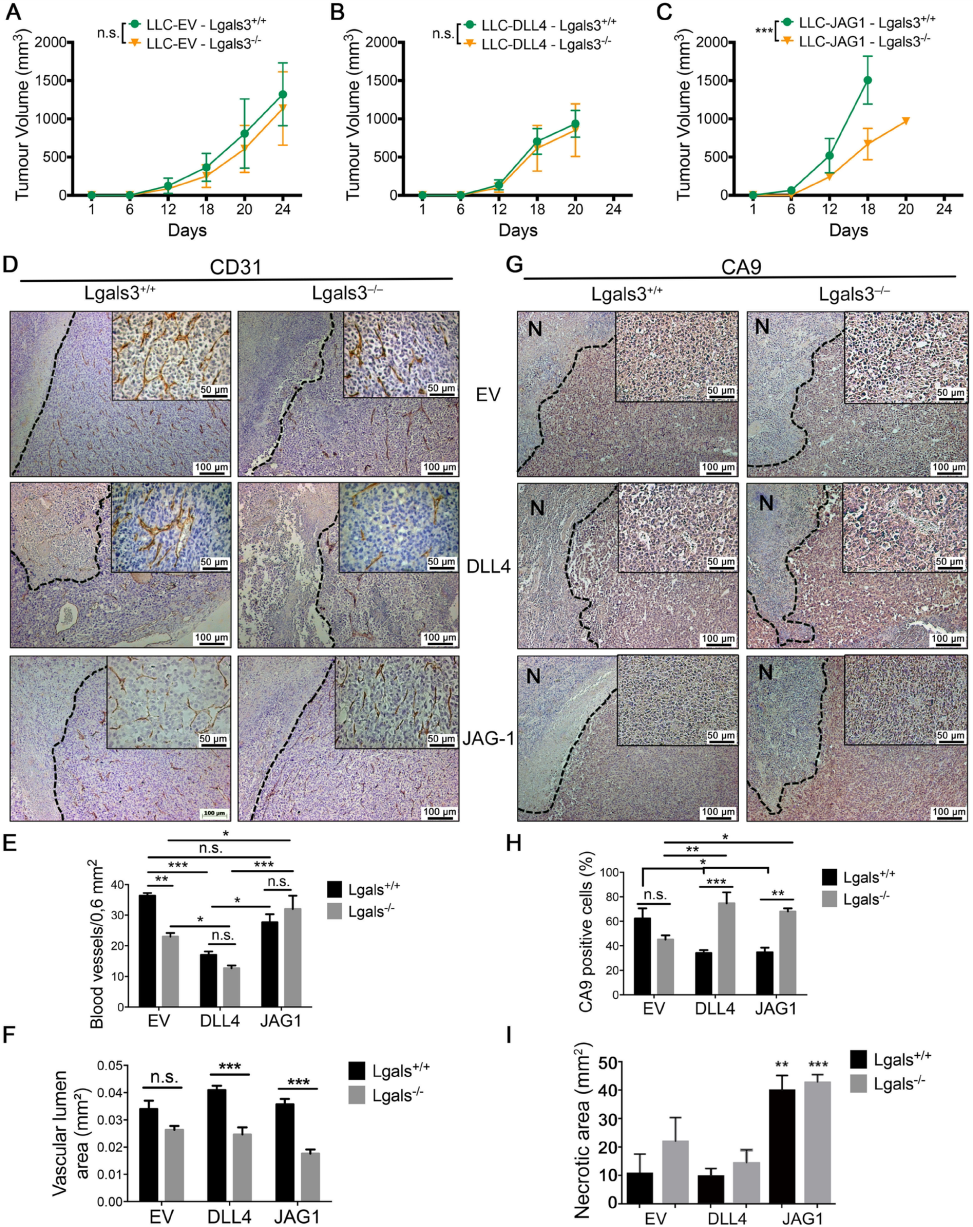
The growth of JAG1 overexpressing tumor is impaired in galectin-3 knock out mice (**A–C**) Tumor growth curve showing the tumor volume of (A) LLC-EV, (B) LLC-JAG1 or (C) LLC-DLL4 cells in C57black/6 Lgals3^+/+^ or Lgals3^−/−^ mice. (**D–F**) (D) Immunohistochemical staining of CD31 and quantification of (E) the number of blood vessels number and (F) the vascular lumen area of LLC-EV, LLC-JG1 or LLC-DLL4 tumors inoculated in C57black/6 Lgals3^+/+^ or Lgals3^−/−^ mice. (**G–I**) (G) Immunohistochemical staining of CA9 and quantification of (H) the percentage of CA9 positive cells and (I) the “N” necrotic area of LLC-EV, LLC-JG1 or LLC-DLL4 tumors inoculated in C57black/6 Lgals3^+/+^ or Lgals3^−/−^ mice. Data are the mean (S.D.), *n* = 3 representative (A–C, E–F and H–I), or are representative microphotograph of immunohistochemical staining (D and G) of three independent experiments. Data analyzed by one-way ANOVA and non-linear regression

Both JAG1 [30, 31] and DLL4 [32, 33] are up regulated in several cancer cells and can control neovascularization and stimulate tumor growth in mice. We tested the ability of gal-3 to interfere with JAG1 and DLL4-induced tumor growth. Lewis lung carcinoma cells (LLC) were transduced with an empty vector retrovirus (LLC-EV), JAG1-encoded retrovirus (LLC-JAG1) or DLL4-encoded retrovirus (LLC-DLL4) (Supplementary Figure 6A and S6B) and implanted subcutaneously into C57black/6 Lgals3^+/+^ or Lgals3^−/−^ mice. No differences in the growths of LLC-EV (Figure 6A) and LLC-DLL4 (Figure 6B) were found between Lgals3^−/−^ mice and Lgals3^+/+^ mice. When LLC-JAG1 was grown in Lgals3^−/−^ mice (Figure 6C), the growth was significantly reduced in comparison to Lgals3^+/+^ mice. Interestingly, there was a more rapid growth of LLC-JAG1 tumors in Lgals3^+/+^ mice at 18 days (1500 mm^3^), compared to LLC-EV and LLC-DLL4 (≈ 500 mm^3^).

At the end of the experiment, tumors were collected for immunohistochemical and mRNA analysis. Although LLC-JAG1 tumors grown in Lgals3^+/+^ mice were significantly bigger than all the other groups (Supplementary Figure 6C), no correlation between the tumor size and Ki67 antigen expression (Supplementary Figure 6D and S6E) was found between groups. After staining tumors for CD31, we found that LLC-EV tumors grown in Lgals3^−/−^ mice had significantly fewer blood vessel numbers per 0.16 mm^2^ than tumors grown in Lgals3^+/+^ mice (Figure 6D and 6E) but similar vessel lumen area (Figure 6F), implying a bigger lumen area per vessel. This is an expected effect of increased activation by DLL4 [34, 35] and a predicted effect of the reduction of gal-3 from our *in vitro* data. No change in hypoxia or necrosis was noted in LLC-EV tumors (Figure 6G–6I). Regarding DLL4, its high expression in tumor cell lines has been reported to reduce vascular density [35, 36]. Accordingly, we found fewer vessels in LLC-DLL4 tumors (Figure 6D and 6E). When LLC-DLL4 tumors were grown in Lgals3^+/+^ mice, the vascular lumen area was increased comparing to Lgals3^−/−^ mice (Figure 6F). This may result in better perfusion and correspondingly, CA9 levels were lower in Lgals3^+/+^ mice (Figure 6G and 6H), although not extending to an increase in necrosis (Figure 6I).

In JAG1-expressing tumors grown in Lgals3^+/+^ mice, there was no change in the vessel number or area, when compared to LLC-EV, which is in contrast to DLL4 tumors (Figure 6D–6F). Surprisingly we found a decrease in CA9 (Figure 6G and 6H) associated with an increase in the necrotic area of 3-4 fold (Figure 6I) in LLC-JAG1 tumors. When grown in Lgals3^−/−^ mice, LLC-JAG1 tumors presented a decrease in lumen area (Figure 6F) and an increase in CA9 (Figure 6G and 6H) with no change in the extensive necrosis (Figure 6I) when compared to Lgals3^+/+^ mice. These results imply that necrosis may not be the main reason for the differential effects of JAG1 in Lgals3^−/−^ mice. However we cannot rule out that, further *in vivo* mechanisms may be responsible for the action of JAG1 besides the clear effects on vasculature. We also observed that gal-3 was expressed in all tumors and its expression was increased close to hypoxic areas (Supplementary Figure 6G). Overall we found that gal-3 increased the growth rate of JAG1-expressing tumors and the vascular lumen area while decreasing the tumor hypoxic areas.

## DISCUSSION

The molecular mechanisms for gal-3-induced angiogenesis described so far include the binding and activation of VEGFR2 in endothelial cells, α_V_β_3_ integrin clustering and activation of signaling pathways that influence bFGF, VEGF and neuron-glial antigen 2 (NG2) angiogenic activities [13–16]. Here we describe a novel mechanism by which gal-3 promotes tumor angiogenesis. Our study suggests that under hypoxic conditions, gal-3 is released by cancer cells and preferentially binds to ECs themselves induced to increase binding by hypoxia. Upon binding to ECs gal-3 triggers angiogenic sprouting by promoting JAG1/Notch signaling activation.

Most of the gal-3 functions can be explained by its capacity to form oligomers in a concentration-dependent equilibrium, which accounts for its ability to crosslink cell surface proteins. Since Notch activation requires JAG1/DLL4 ligands expressed on a signal-sending cell to interact with Notch receptor on a neighboring signal-receiving cell (*trans*-interaction) [37], we can hypothesize that gal-3 may help bring receptor and ligand together, thus increasing Notch signaling. Indeed, gal-3 is known to behave as a monomer in concentration < 50 nM [38]. While in this study we used 37 nM of rhgal-3, we cannot disregard that HUVECs already display some gal-3 on the cell surface (data not shown), which may have led to oligomer formation through self-association on the cell surface. In the serum of cancer patients, gal-3 concentration levels can range from 20–620 ng/mL (0.74–22 nM) in breast cancer; 20–950 ng/mL (0.74–35 nM) in gastrointestinal tumors; 20–807 ng/mL (0.74–29 nM) in lung cancer; and 35–366 ng/mL (1.3–12.4 nM) in ovarian cancer [39]. At higher concentrations >25 μg/mL (> 925 nM) gal-3 is described to multimerize, creating a rigid structure called a lattice, which restrains the lateral movement of membrane glycoproteins [38] and can result in cell adhesion inhibition [40, 41]. Thus, a high concentration of gal-3 might restrain Notch receptor interaction with the ligands present on an opposite cell inhibiting Notch signaling.

Our data also demonstrated that Notch signaling activation by gal-3 was independent of VEGFR2. This is of particular importance since most antiangiogenic therapies are designed to disrupt VEGF-VEGFR interactions [42]. Our result suggests the induction of a compensatory angiogenic pathway by hypoxia that would induce gal-3 and activate signaling via the JAG1/Notch pathway, which may contribute to limit the efficacy of anti-VEGF treatment [43].

Indeed, it is known that tumor hypoxia can activate compensatory signaling pathways associated with glycosylated ECs receptors in response to VEGF blockade [44]. Both gal-3 and JAG1 are overexpressed in several malignancies such as prostate cancer, breast cancer, glioma and head and neck cancers and are largely appreciated as potential targets for cancer therapy, including antiangiogenic therapy [45, 46]. JAG1-induced Notch signaling plays an important role in tumor biology affecting both proliferation and metastasis of tumor cells and also activating neighboring endothelial cells to promote neovascularization and growth of experimental tumors in mice [47, 48]. Similarly, gal-3 has been associated with tumor growth and cell invasion [49, 50]. Therefore, circumstances that lead to the up-regulation of gal-3 in the tumor microenvironment may influence JAG1 activity both in cancer and endothelial cells. For instance tumor necrosis factor-α (TNF-α), a proinflammatory cytokine, was found to induce the expression of gal-3 [51] together with JAG1 [24].

JAG1 has been identified as a critical component in the process of tip cell selection, and, unlike DLL4 it has been considered as proangiogenic [5, 24]. Loss of JAG1 has been seen to significantly decrease the number of tips and filopodia as well as EC proliferation. On the other hand, JAG1 overexpression increased vessel branching, EC density and downregulated DLL4-Notch signaling decreasing *Hes-1* and *Hey-1* levels [4]. Here we reported that extracellular gal-3 phenocopied the role of JAG1 in angiogenesis by increasing HUVEC proliferation and the tip cell number. Moreover, in the presence of gal-3, JAG1 ligand but not DLL4 present on HUVECs was able to induce Notch signaling activation in murine endothelial receiving cells (sEnd-1). Additionally, by SPR, we found that JAG1 presented 5 times more gal-3 binding-sites than DLL4. Accordingly, using bioinformatics prediction tools, our results consistently revealed that JAG1 presents more potential N- and O-glycosylation sites than DLL4 protein. Although not every potential glycosylation site predicted may occur and be recognized by gal-3, gal-3 can bind to both N- and O-glycans present on glycoproteins [52]. Since JAG1 is a larger protein than DLL4 displaying more glycan structures than DLL4, we suggest that gal-3 may facilitate and switch on JAG1/Notch-1 signaling in endothelial cells. Therefore, our data suggest that the proangiogenic functions of extracellular gal-3 might be mediated by the activation of JAG1/Notch-1 signaling in endothelial cells.

Previously, another mechanism of regulation of the switch between DLL4 and JAG1 signaling was proposed whereby the glycosyltransferase fringe modified Notch receptors to enhance DLL4/Notch signaling in endothelial cells. As a consequence, fringe modification reduces Notch activation upon Jagged1 binding [4]. Our results may contribute directly to this model if modification by fringe changes gal-3 binding properties, although our work using recombinant JAG1 and DLL4 shows such modification of the ligands is not needed for the effects of gal-3 on endothelial cells.

Although not yet explored, gal-3 and JAG1/Notch signaling play similar roles in multiple cellular processes such as immune system regulation and allergic inflammation [19, 53–55]. Additionally, both gal-3 and JAG1 are overexpressed in several malignancies and are largely appreciated as potential targets for cancer therapy, including antiangiogenic therapy [45, 46]. Thus, our results may have a wider significance that extends to other cell types and biological processes that are regulated by the Notch signaling pathway

In this study we found that gal-3 has an essential role in regulating the tumor growth of JAG1 expressing tumors. We observed that in Lgals3^−/−^ mice, LLC-JAG1 cells had a significant reduced growth rate compared to LLC-JAG1 grown in Lgals3^+/+^ mice while DLL4 and EV-derived tumors were not affected. Since JAG1 can increase cancer progression by its pro-proliferative functions [47, 56], our findings suggest that JAG1's role in cancer progression can be regulated by microenvironmental gal-3. Additionally, we identified that tumors grown in Lgals3^−/−^ mice presented increased hypoxic areas in comparison to Lgals3^+/+^ mice and the reverse was true for the vascular lumen area, suggesting impaired blood vessel formation in the absence of microenvironmental gal-3. Additionally, JAG1-expressing tumors presented higher CD31 mRNA levels when in the presence of microenvironmental gal-3. Taking into account all these results, we suggest that gal-3 positively regulates JAG1's biological role in inducing tumor growth, endothelial sprouts, blood vessel maturation and the formation of luminal structures [57, 58]. Thus, and in agreement with our *in vitro* findings, microenvironmental gal-3 seems to regulate vessel formation in tumors via modulation of JAG1/Notch signaling.

The *in vivo* effects of JAG1 and DLL4 were more complex than the *in vitro* ones. Indeed, JAG1 is recognized to induce inflammation in blood vessels and immune activation [59]. Moreover, inhibition of JAG1 protects from atherosclerosis [60]. Therefore, a more detailed analysis of blood flow *in vivo*, JAG1/DLL4 distribution in the tumors, as well as the evaluation of vascular differentiation, proliferation and leakiness will be needed to elucidate the *in vivo* mechanisms of JAG1 and DLL4. Still, such studies are beyond the scope of this paper. However it is well recognized pathologically that necrosis is a sign of aggressive tumor biology, often associated with angiogenesis and macrophage infiltration [61]. We hypothesize that these effects may also contribute to JAG1 *in vivo* biology, and clearly to a more aggressive phenotype induced alongside with vascular changes. The latter seem important for tumor growth as growth is reduced in Lgals3^−/−^ mice, even with an increase in necrosis but reduction in the tumor vasculature. Moreover, in our *in vivo* study we particularly examined the role of host gal-3 in tumor growth and angiogenesis. Though, LLC tumor cells also produce gal-3 (data not shown) and therefore the *in vivo* effects observed in Lgals3^−/−^ mice may be rescued by this source.

**Figure 7:**
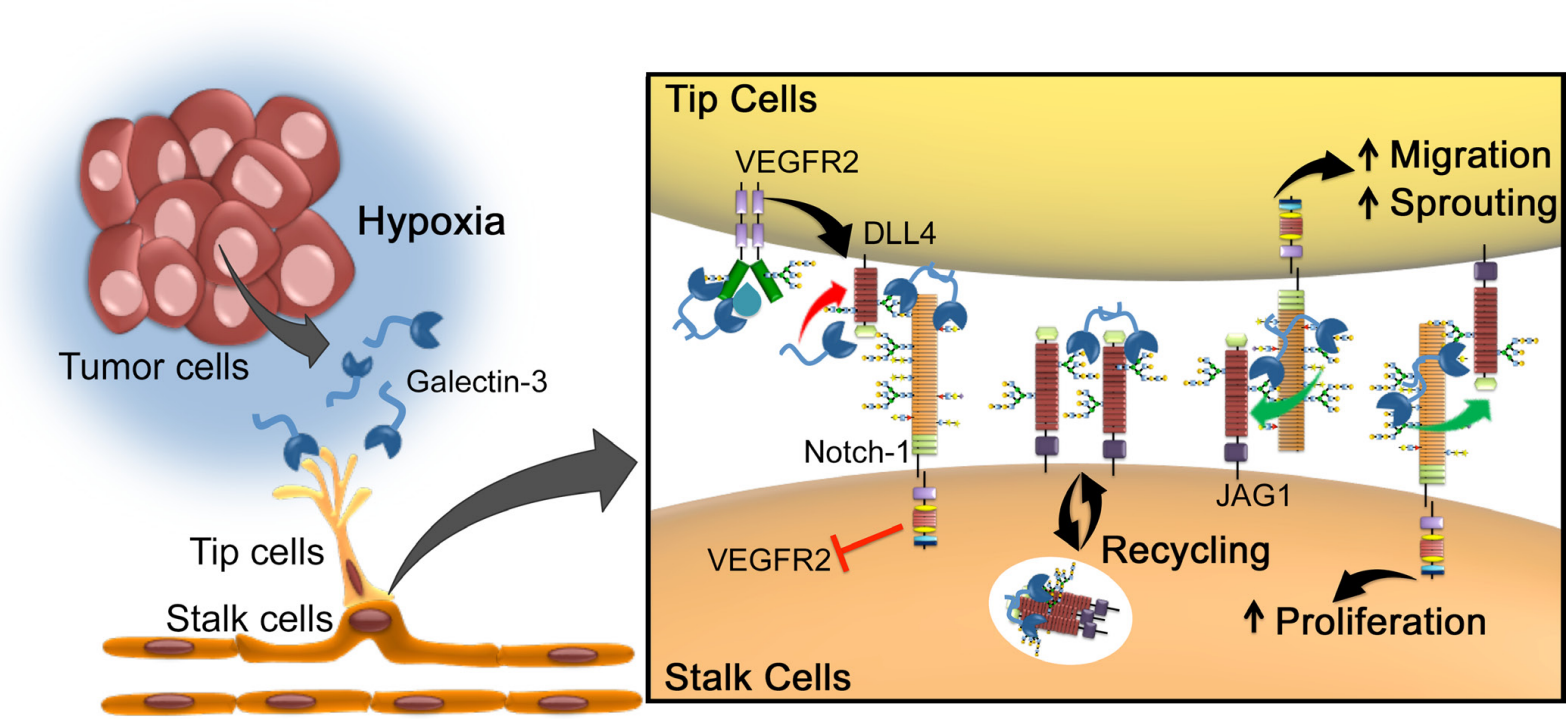
Proposed model for galectin-3-mediated angiogenesis via JAG1/Notch signaling Tumor environmental signals such as hypoxia stimulates cancer cells to release the proangiogenic gal-3, in order to increase the formation of new blood vessels. Due to changes in the glycosylation status triggered by hypoxia, gal-3 secreted by cancer cells binds to endothelial cells and activates glycan-dependent circuits, including activation of VEFGR2 and the stabilization of JAG1 over DLL4. Once sprouting begins, while VEGFR2 induces the expression of DLL4 in endothelial tip cells, gal-3 limits DLL4-Notch signaling by decreasing DLL4 protein and increasing JAG1/Notch interaction. JAG1/Notch signaling then induces (1) migration of tip cells and (2) proliferation of stalk cells. This mechanism is in contrast to another mechanism of regulating the ratio of DLL4 over JAG1 signaling via fringe, but both processes may be involved [4]. Mechanistically, galectin-3 can modulate this highly dynamic process: (1) by interfering with DLL4 and JAG1 ratio; (2) enhancing Notch-ligand interaction and consequently Notch signaling activation; (3) inducing JAG1 endocytosis in order to make it a more effective ligand; or (4) stabilizing JAG1 on the cell surface making it more available to interact with Notch receptor

We propose a new mechanism by which tumor released-gal-3 increases angiogenesis by influencing JAG1/Notch signaling in endothelial cells (Figure 7). Indeed, since JAG1 and DLL4 have opposing roles in angiogenesis, the identification of molecules that can change the ratio and function of JAG1 and DLL4 might impact the regulation of sprouting angiogenesis [4]. In this context, the coordinated expression of JAG1 and DLL4 regulated by gal-3 seems to be a key determinant to functional neovascularization. Therefore, targeting gal-3 may open novel perspectives to interfere with angiogenesis and may provide an experimental strategy to overcome anti-VEGF therapy and hypoxia-induced resistance.

## MATERIALS AND METHODS

### Detailed methods are provided in Supplemental Data

#### Cell culture

HUVECs (Lonza) were cultured in EGM-2 media supplemented with growth factors and 5% of fetal bovine serum (FBS) as provided in the EGM-2 Bullet Kit (Lonza). The cells between passages 2 and 6 were used. LLC (ATCC CRL-1642), MCF7 (ATCC HTB-22), MDA-MB-231 (ATCC HTB-26) tumor cells and sEnd-1 cell line (murine endothelial cells transformed by middle T antigen of polyoma virus [62]), were cultured in Dulbecco modified Eagle medium (DMEM, Gibco) supplemented with 10% of fetal bovine serum (FBS, Gibco). For virus production, HEK293t cells (ATCC CRL-3216) were used and cultured in DMEM, supplemented with 10% FBS. Mycoplasma contamination in cultured cells was excluded by using Lonza Mycoplasma Detection Kit.

### JAG1 and DLL4 ELISA binding assay

1μg of the recombinant human JAG1 or DLL4 were diluted in PBS, and incubated overnight in a 96-well plate. The next day, the plate was washed and blocked with 1% BSA/PBS and 1μg of recombinant human gal-3 or gal-3C in the presence or absence of lactose (5 mM) and sucrose (5 mM) was added to the plate. Galectin-3 binding was detected after addition of a detection antibody according to the manufacturer's procedure (ELISA galectin-3 Duo Set, R&D Systems). Detection assay: (1) without rhgal-3 or rhgal-3C; (2) without the primary antibody; or (3) blank, were used as a negative control.

### JAG1 and DLL4 SPR binding assay

SPR experiments were performed on a BIAcore T200 instrument at 37°C in filtered buffer HBS-P pH 7.4. Proteins (rhDLL4 and rhJAG1) were immobilized on a Series S CM5 chip (GE Healthcare) using GE Healthcare's amine coupling kit. Reference flow channels underwent a cycle of activation and deactivation but were left empty. Serial dilutions of FPLC purified monomeric rhgal/ rhgal3C in HBS-P buffer were used as analyte, with or without addition of 50 mM lactose or 50mM sucrose. Curve fitting was performed in GraphPad Prism 6.0.

The number of exposed gal-3 binding sites per immobilized molecule of JAG1 or DLL4 was calculated using the equation:

$$bindings\;sites\;per\;immobilized\;molecule=\frac{RU_{\max}/MW_{analyte}}{RU_{\text{immobilized}}/MW_{ligand}}$$

(Where RU_max_ is the maximum binding and MW is molecular weight) [63].

### Gene expression analysis

Total RNA from cell cultures was isolated with Tri-Reagent (Sigma) according to the manufacturer's instructions. Complementary DNA (cDNA) was synthesized using the High capacity cDNA RT kit (Applied Biosystems), according to the manufacturer's protocols. Quantitative PCR analysis was performed in triplicate using the SensiMix SYBR No-ROX kit (Bioline). Relative quantification was done using the ΔΔCt method normalizing to GAPDH gene expression (See Supplementary Table 1 for primers details).

### Prediction of N-linked and O-linked glycosylation profile for JAG1 and DLL4 proteins

NetNGlyc 1.0 server [26] was used to find the potential N-glycosylation sites for JAG1 (PubMed accession number: U73936) and DLL4 (PubMed accession number: AF253468) proteins. NetNGlyc 1.0 server (http://www.cbs.dtu.dk/services/NetNGlyc/) predicts N-glycosylation sites in human proteins using artificial neural networks that examine the sequence context of AsnXaaSer/Thr sequences. Only predicted N-glycosylation sites with a potential score > 0.5 were considered.

NetOGlyc 4.0 server (http://www.cbs.dtu.dk/services/NetOGlyc/) [27] was used to find the potential N-glycosylation sites for JAG1 and DLL4 proteins. The NetOglyc server produces neural network based predictions of mucin type GalNAc N-glycosylation sites in mammalian proteins. Only Gscore > 0.5 were predicted to be O-glycosylated.

### Sprouting angiogenesis assay

HUVEC spheroids (500 cells) were generated by the hanging-drop method [64]. Briefly, HUVECs were harvested and suspended in culture medium containing 0.20% (wt/vol) methylcellulose and seeded on the inside face of the lid of a 150 mm culture dish (Corning). 16 h–24 h later, spheroids were collected and embedded into 500 μL of 2,5 mg/mL fibrinogen solution (Sigma) containing 0,15 Units/mL of aprotinin (Sigma). After polymerization, spheroids were cultured for 24 h in the presence of EGM2 medium and growth supplements. In a set of experiments, after polymerization, MCF7 scramble and galectin-3 silenced cells were cultured on the top of the gel and spouting angiogenesis was evaluated after 24 hrs. In another set of experiments, HUVEC spheroids were generated after labeling half of cells with a viable fluorescent green dye and the other half with a viable fluorescent red dye according to manufacturer's protocol (Cell Tracker^TM^ Green CMFDA and Cell Tracker^TM^ Red CMPTX dyes from Invitrogen). Before mixing red and green-labeled HUVECs in a 1:1 ratio, rhgal-3 (37 nM) was added or not to red-labeled HUVECs. Spheroids were then embedded into 200 μl of fibrinogen solution and plated on a glass-bottom dishes (MatTek). After 24 h, spheroids were fixed overnight at 4°C in 4% formaldehyde/PBS and pictures of the sprouts were taken using an inverted microscope (Zeiss Axiovert 200M). The number of tip cells of each color was counted. Alternatively, 24 h after transfection of 3 different siRNA against JAG1 or DLL4 or scrambled, HUVEC spheroids were generated.

All experiments were performed at least three times and in triplicate. Angiogenesis was quantified by counting the number of sprouts and the length of sprouts for each spheroid (∼30–50 spheroids) per condition, and analyzed with the ImageJ software.

### Determination of JAG1 and DLL4 half-life

To assess protein stability, HUVECs were incubated with 37 nM of gal-3 for 15 mins. After that period, the growth medium was replaced with fresh medium containing 0.1 mM of cycloheximide to stop protein biosynthesis. Cells were treated for 0 to 8 hrs and then collected for Western blotting. ImageJ software was used to quantify western blot bands.

### Western blotting

Cells were lysed in RIPA buffer and 50μg of proteins were separated by Novex NuPAGE SDS-PAGE gel system (Invitrogen) and then transferred overnight to a PVDF membrane (Invitrogen). The membrane was incubated with anti-gal-3, anti-cleaved Val1744 (NICD1), anti-JAG1, anti-DLL4, anti-Notch1, anti-VEGFR2, anti-VEGFR-p and HIF1-α. Anti-β-actin-peroxidase was used as a loading control. Horseradish peroxidase (HRP)-conjugated secondary antibodies were detected using the enhanced chemiluminescence (ECL) reagent (GE Healthcare). Image J software was used to analyze the densitometry value of Western blots bands. Antibody sources are listed in Supplemental Data.

### Treatment of HUVECs with MCF7 cells conditioned medium

Scrambled or shRNA-Gal-3 MCF7 cells were cultured for 48 hrs. After this period, the conditioned medium was incubated with HUVECs (10^6^) for 6 hrs and mRNA levels were analyzes. Galectin-3 from the conditioned medium was quantified by ELISA (ELISA galectin-3 Duo Set, R&D Systems) according to manufacturer instructions.

### Generation of JAG1 or DLL4 overexpressing HUVECs

Stable HUVEC control or overexpressing the full length JAG1 or DLL4, were generated after co-transfection of 30 μg of empty vector-containing pLenti6.2-V5 plasmid or full length JAG1 cDNA-containing pLenti6.2-V5 plasmid or full length DLL4 cDNA-containing pLenti6.2-V5 plasmid with 15 μg pPAX2 and 5μg of pMDG.2 (Addgene) into HEK293t packaging cell line using the CaCl_2_ method. The viral supernatant was recovered and the transduced cells were generated by infection with 5 MOI (multiplicity of infectious units) of lentiviral particles. On the next day, cells were replaced with fresh medium, and a day later, cells were selected with 5 μg/mL of Blasticidin for 1 week. JAG1 and DLL4 expression was confirmed by Western blot and qPCR.

### *In vivo* studies

Lgals3^+/+^ and Lgals3^−/−^ mice were bred at the animal facility of the Faculty of Medicine from the University of São Paulo (FMUSP), and all experiments complied with the relevant laws and were approved by local animal ethics committees (protocol approval 147/11). Empty vector (LLC-EV), murine JAG1 overexpressing (LLC-JAG1) or murine DLL4 overexpressing (LLC-DLL4) Lewis Lung carcinoma cells (LLC) were kindly provided by Dr Ji-Liang Li (University of Oxford, UK). Briefly, the murine Lewis Lung carcinoma cells (LLC) were transduced with retrovirus containing full-length mouse JAG1 or DLL4 or empty vector as previously described [65]. Retroviral supernatants were prepared using the bicistronic pLZRS-IRES-GFP plasmid with the Phoenix amphotropic packaging cell line. Six- to 8-week-old male WT or Lgals3^−/−^ C57BL/6 mice were implanted subcutaneously with 10^6^ LLC-EV or LLC-JAG1 or LLC-DLL4 cells. Each group consisted of five mice. When tumors reached the size 1–1.5 cm^3^ volume, mice were sacrificed and tumors were excised for mRNA extraction and immunohistochemistry analysis.

### Immunostaining

Tissue sections were deparaffinized in xylene and rehydrated in serial alcohol dilutions. Tissue sections were stained with anti-CA9 antibody (sc-17253, Santa Cruz) or anti-CD31 (DIA-310-M, Dianova), followed by a secondary anti-goat or anti-rat biotinylated antibody (Vector Laboratories), respectively. Next, streptavidin-peroxidase (Sigma) was added and color development was done with DAB (DAKO). Nuclei were counterstained with hematoxylin. Lumen areas were identified by the presence of CD31-positive vessel-like structure with a lumen. Then lumen areas were traced through freehand selections on digital images and measured with the NIH ImageJ software. The number of vessels was counted and calculated as vessels per square-millimetre using ImageJ software. CA9 staining was quantified by the percentage of positively staining nuclei using the TMARKER software at a magnification of 200-fold [66]. Three or more fields per animal were analyzed and averaged. Averages for 3 or more animals per group were compared.

### Statistical analysis

No statistical method was used to predetermine sample size. *In vitro* experiments were performed at least in triplicate with at least three replicates per experiment. *Invivo* experiments were performed at least in duplicate with 5 mice per treatment group. Mice or samples were randomly assigned to various groups. No blinding of investigators was done. Statistical analyses were performed using GraphPad Prism 6.0 software (GraphPad Software, Inc.). Results are shown as means ± standard deviation (S.D.). To determine statistically significant differences between groups, normal distribution was assumed and unpaired Student's *t-test*, one-way or 2-way analysis of variance (ANOVA) were used. For xenograft studies, the growth rates were calculated by non-linear regression (exponential growth model). The variance was determined by *F*-test. *P*-values less than 0.05 were considered significant.

## SUPPLEMENTARY MATERIALS TABLE AND FIGURES


