# Supplementary material for: Galectin-3 acts as an angiogenic switch to induce tumor angiogenesis via Jagged-1/Notch activation
Source: Oncotarget. 2017 May 9;8(30):49484–501. doi: 10.18632/oncotarget.17718 (PMC5564783; doi:10.18632/oncotarget.17718)
Supplement: Supplementary file 1 [file oncotarget-08-49484-s001.pdf]

Galectin-3 acts as an angiogenic switch to induce tumor angiogenesis via Jagged-1/Notch activation

SUPPLEMENTARY MATERIALS

SUPPLEMENTARY METHODS

Reagents

Recombinant human Jagged-1-His tagged protein (rhJAG1) was purchased from Sino Biological and recombinant human Delta-like-4-His tagged (rhDLL4) was obtained from R&D Systems. The VEGFR inhibitor Vatalanib (Selleckchem) was dissolved in water and used at a concentration of 100 nM. The γ-secretase inhibitor dibenzazepine (DBZ) (Calbiochem) was dissolved in dimethyl sulfoxide and used at a concentration of 10 nM. Cycloheximide (Sigma) was dissolved in water and used at a concentration of 100 nM. Biotinylated lectins *Erythrina cristagalli* (ECA), *Phaseolus vulgaris* (L-PHA), *Sambucus nigra* (SNA) and *Maackia amurensis* (MAL) were purchased from Vector Laboratories. *Arachis hypogaea* (PNA) was purchased from Sigma. Anti-galectin-3 antibody was produced with M3/38 hybridoma (M3/38, ATCC TIB166). Anti-CA9 (sc-17253) was from Santa Cruz. Anti-cleaved Notch-1 at Val1744 (#4147), anti-JAG1 (#28H8), anti-DLL4 (#2589), anti-Notch-1 (#D1E11), anti-VEGFR2 (#2479), anti-VEGFR2-p (#3770), and anti-HIF1-α (#D2U3T) antibodies were from Cell Signaling; anti-β-actin-peroxidase (A3854) from Sigma-Aldrich. Anti-CD31 (DIA-310-M) was from Dianova. Horseradish peroxidase (HRP)-conjugated secondary antibodies were purchased from DAKO. Biotinylated secondary antibodies anti-goat (BA-5000), anti-rat (BA-4001) and anti-rabbit (BA-1100) were from Vector Laboratories. Anti-rabbit-Alexa-488 (A-11034) and anti-goat-Alexa-546 (A-11056) were from Invitrogen. Streptavidin-Cy5 (SA1011) was from Invitrogen. DyLight-488 mAb labelling kit (catalogue no. 53025) was purchased from Thermo Fisher Scientific.

Recombinant human galectin-3 and galectin-3C preparation

Galectin-3 and galectin-3C were produced in *Escherichia coli* Bl21/DE3 containing the pET11a plasmid with the human galectin-3 (rhgal-3) coding DNA or the C-terminal domain fragment of galectin-3 (rhgal-3C) (gift from Dr. Nozomu Nishi, Kagawa University, Japan) and purified by affinity chromatography on lactosyl-Sepharose (Sigma) as previously described [1]. Lactose was removed by gel-filtration chromatography on desalting columns and contaminating LPS was eliminated by affinity chromatography on detoxi-gel beads (Pierce). The endotoxin levels were less than 0,1 EU per µg of protein as determined by the LAL method (Lonza). Conjugation of rhgal-3 with DyLight488 was performed accordingly to manufacturer's instructions.

Gene expression analysis

Total RNA from tumor tissue was isolated with Tri-Reagent (Sigma) according to the manufacturer's instructions. Complementary DNA (cDNA) was synthesized using the High capacity cDNA RT kit (Applied Biosystems), according to the manufacturer's protocols. Quantitative PCR analysis was performed in triplicate using the SensiMix SYBR No-ROX kit (Bioline). Expression values were normalized to the geometrical mean of housekeeping genes GAPDH, HPRT and β-actin and fold-changes between were determined by the ΔΔCt method [2].

CyQuant proliferation assay

HUVECs proliferation in response to rhgal-3 was performed by plating cells in quadruplicate (2000 cells/well) in 96-well plates, followed by incubation with CyQUANT (Invitrogen) as described by manufacturer.

Flow cytometry

For flow cytometry, MCF7, MDA-MB-231 and HUVEC cells were harvested and $1x10^6$ cells were incubated with 1% BSA/PBS for 1 hour in at 4^oC. Next, cells were incubated with 1µg of the biotinylated lectins MAL-II, PNA, ECA, SNA or L-PHA for 1 hour. Subsequently cells were washed with PBS, and biotinylated lectins were detected with streptavidin-Cy5 for 45 min. After final washing, cells were fixed with 4% paraformaldehyde. Alternatively, cells were incubated with different doses (37 nM, 370 nM, 925 nM, 1.85 µM) of DyLigth 488 labelled-hrGal-3 or DyLigth 488 labeled-hrGal-3C in the presence or absence of lactose (50mM) for 1 hour and fixed in 4% paraformaldeyde. Analysis was made using the flow cytometer CyAn™ ADP Analyzer from Beckman Coulter. Data were subsequently evaluated with FlowJo vX 0.7 software.

Generation of MCF7 galectin-3 knockdown cells

Stable shRNA MCF7 cell line targeting galectin-3 (TRCN0000029305, Sigma) and negative control (SHC016, Sigma) were generated after co-transfection of 30 µg of shRNA-containing plasmids with 15µg pPAX2 and 5 µg of pMDG.2 (Addgene) into HEK293t packaging cell line utilizing $CaCl_2$ method. The viral supernatant was recovered and the transduced cells were generated by infection with 2 MOI (multiplicity of infectious units) of shRNA lentiviral particles. On the next day, cells were replaced with fresh medium, and a day later, cells were selected with 2 µg/mL of puromycin for 1 week. Galectin-3 knockdown (shRNA-gal-3) and negative control (scramble) cells were generated and subjected to Western blot and mRNA quantification to investigate galectin-3 expression.

JAG1 and DLL4 siRNA transfection

HUVECs were transfected at 60% of confluency with JAG1 or DLL4 specific siRNAs and negative control (scramble) (Ambion, Life Technologies) at a final concentration of 20 nM using RNAiMAX reagent (Invitrogen) in Opti-MEM I reduced serum medium (Invitrogen). After 6 hrs, transfection medium was replaced by complete EGM-2 medium. 24 hrs after transfection, spheroids were generated. Cells were collected for western blot and Real-time analysis after 48 hrs of inhibition.

Short interfering RNAs (siRNAs) sequences were: human JAG1 (1) Sense: 5′-CGUUCAACCUGACAGUA UTT-3′, Antisense: 5′-AUACUGUCAGGUUGAACGG TG- 3′; human JAG1 (2): Sense: 5′-CGAUUAUUGUGAG CCUAAUTT-3′, Antisense: 5′-AUUAGGCUCACAAUA AUCGAT-3′; human JAG1 (3): Sense: 5′-CCUCAUCCCU GUUACAACATT-3′, Antisense: 5′-UGUUGUAACAGG GAUGAGGGC-3′; human DLL4 (1): Sense: 5′-CCCUAG CUGUGGGUCAGAATT-3′, Antisense: 5′-UUCUGACC CACAGCUAGGGAG-3′; human DLL4 (2): Sense: 5′-GG UACCUUCUCGCUCAUCATT-3′, Antisense: 5′-UGAUG| AGCGAGAAGGUACCCG-3′; human DLL4 (3): Sense: 5′-CAACUGCCCUUCAAUUUCATT-3′, Antisense: 5′-UG AAAUUGAAGGGCAGUUGGA-3′.

Immunofluorescence

HUVECs in monoloayer or HUVECs spheroids were treated with rhgal-3 for 6 or 24 hrs, respectively. After this period, samples were fixed in paraformaldehyde 4% and incubated with anti-JAG1 (Cell signaling, #2H8H) and anti-DLL4 (Santa cruz, sc-18640) antibodies and detected with anti-rabit-Alexa-488 (Invitrogen) or anti-goat-Alexa-546 (Invitrogen), respectively. Nuclei were stained with DAPI (4’,6-diamidino-2-phenylindole). Pictures were taken using Zeiss 780 Inverted Confocal and ImageJ was used to quantify the number of tip cells and fluorescence intensity.

Cell surface protein isolation

The Pierce cell surface protein isolation kit (Pierce, Rockford, Illinois, USA) was used for the isolation of cell surface protein and intracellular protein fractions, according to the manufacturer’s protocol with some modifications. Briefly, four 75-cm^2 tissue culture flasks (Costar) at 80% to 90% confluency were incubated with 1 vial of Sulfo-NHS-SS-Biotin (Pierce) dissolved in phosphate-buffered saline (PBS) at 4°C for 30 min, washed with PBS, collected by gentle scraping and pooled and pelleted by centrifugation (at 500 × g for 3 min). Cells were then lysed in 500 ml of lysis buffer (Pierce) supplemented with 1 tablet of the complete protease inhibitor cocktail (Roche, Mannheim, Germany) and incubated on ice for 30 min. Cell lysates were clarified by centrifugation at 4°C (10,000 × g for 10 min) and resulting supernatants were incubated with NeutrAvidin Protein beads (Pierce) at 4°C for 2 h. The beads were collected by centrifugation and washed three times with Wash Buffer (Pierce). Proteins were eluted from the beads by incubation of beads with 450µL of SDS-PAGE Sample Buffer containing 50 mM of dithiothreitol and heat blocking for 5 min at 95ºC. The eluted proteins represented the cell surface fractions. The remaining unbound cell lysates were also collected and represented the intracellular fractions.

Immunostaining

Tissue sections were deparaffinized in xylene and rehydrated in serial alcohol dilutions. Tissue sections were stained with rabbit anti-Ki67 antibody (Vector Laboratories) or anti-galectin-3 antibody (M3/38, ATCC TIB166), followed by a secondary anti-rabbit or anti-rat biotinylated antibody (Vector Laboratories). Next, streptavidin-peroxidase (Sigma) was added and color development was done with DAB (DAKO). Nuclei were counterstained with hematoxylin. Ki-67 staining was quantified by the percentage of positively staining nuclei using the TMARKER software at a magnification of 200-fold [3]. Three or more fields per animal were analyzed and averaged. Averages for 3 or more animals per group were compared.

REFERENCES

1. Hsu DK, Zuberi RI, Liu FT. Biochemical and biophysical characterization of human recombinant IgE-binding protein, an S-type animal lectin. The Journal of biological chemistry. 1992; 267:14167–14174.
2. Livak KJ, Schmittgen TD. Analysis of relative gene expression data using real-time quantitative PCR and the 2(-Delta Delta C(T)) Method. Methods. 2001; 25:402–408.
3. Schuffler PJ, Fuchs TJ, Ong CS, Wild PJ, Rupp NJ, Buhmann JM. TMARKER: A free software toolkit for histopathological cell counting and staining estimation. Journal of pathology informatics. 2013; 4:S2.

Supplementary Table 1: Primer's sequences

Primer	Forward 5′–3′	Reverse 5′–3′
hGAPDH	AGCCACATCGCTCAGACAC	GCCCAATACGACGAAATCC
hJAG1	GGAACCTAGAGCCGAACTCA	AGCCTTGTCGGCAAATAGC
hHEY-1	CGAGCTGGACGAGCCCAT	GGAACCTAGAGCCGAACTCA
hHEY-2	GTACCATCCAGCAGTGCATC	AGAGAATTCAGGGCATTT
hDELTA-4	CCCTGGCAATGTACTTGTGAT	TGGTGGGTGCAGTAGTTGAG
hHES-1	AGTGAAGCACCTCCGGAAC	CGTTCATGCACTCGCTGA
hGAL-3	TGTTTGCAATACAAAGCTGGA	GCAACCTTGAAGTGGTCAGG
mHPRT	CCTCCTCAGACCGCTTTTT	AACCTGGTTCATCATCGCTAA
mGAPDH	TGAGCCTCCTCCAATTCAAC	TTTGTCTACGGGACGAGGAA
mACTIN	CTAAGGCCAACCGTGAAAAG	ACCAGAGGCATACAGGGACA
mHEY-1	CATGAAGAGAGCTCACCCAGA	CGCCGAACTCAAGTTTCC
mHES-1	ACACCGGACAAACCAAAGAC	CGCCTCTTCTCCATGATAGG
mHEY-L	CTGAATTGCGACGATTGGT	GCAAGACCTCAGCTTTCTCC
mHEY-2	GTGGGGAGCGAGAACAATTA	GTTGTCGGTGAATTGGACCT
mCD31	CGGTGTTCAGCGAGATCC	ACTCGACAGCATGGAAATCAC

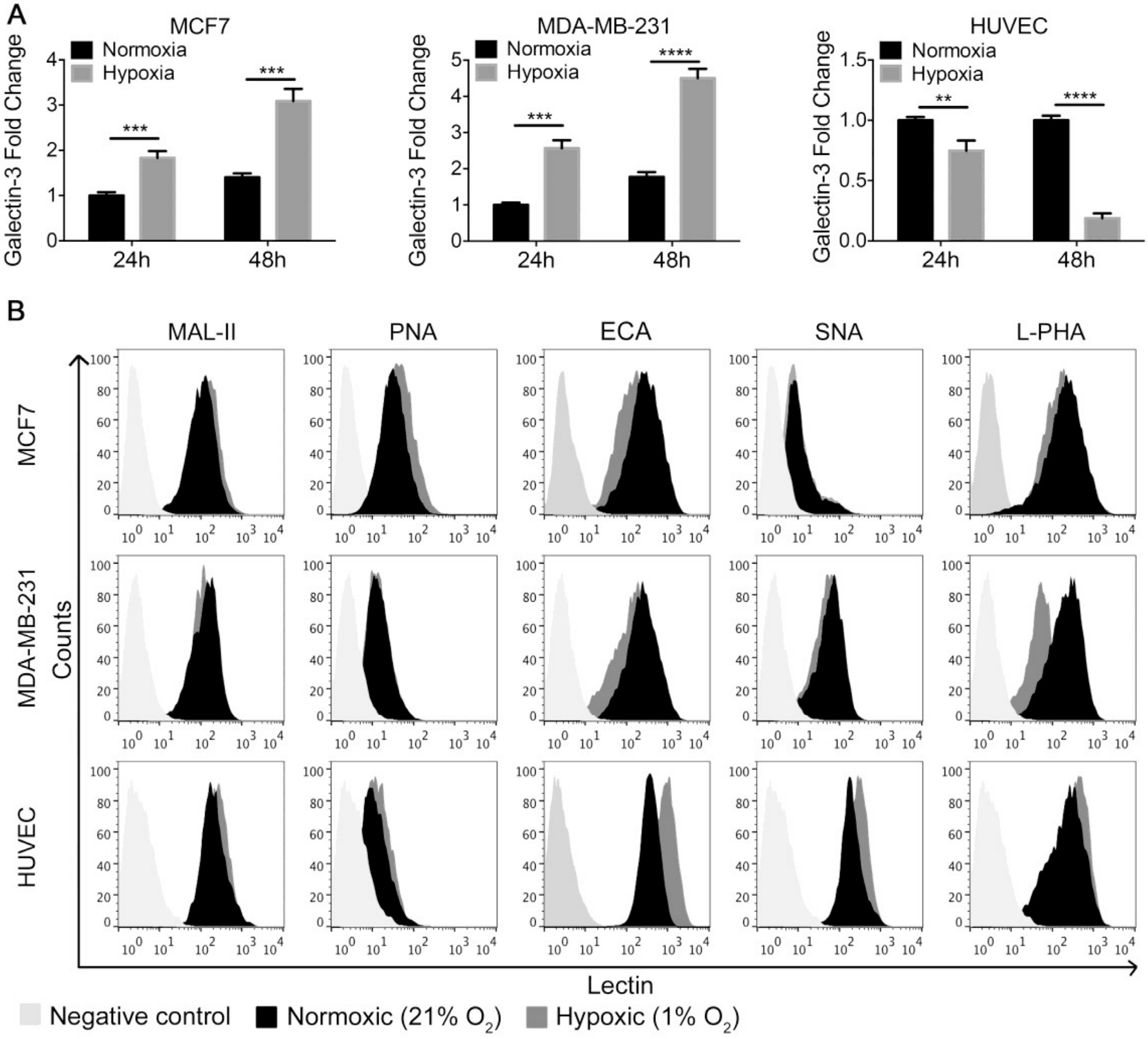

Supplementary Figure 1: Tumor-secreted galectin-3 under hypoxic conditions increases it's binding to endothelial cells. (**A**) MCF7, MDA-MB-231 and HUVECs cells were cultured for 24 or 48 hrs cultured under normoxic (21% O_2) or hypoxic (1% O_2) conditions. After this period, total RNA was extracted and galectin-3 mRNA levels were evaluated by Real-Time PCR. Relative quantification was done using the ΔΔCt method normalizing to GAPDH gene expression. (**B**) MCF7, MDA-MB-231 and HUVEC were cultured under normoxic or hypoxic conditions. After 48 hrs, cells were collected and incubated with biotinylated lectins MAL-II, PNA, ECA, SNA and L-PHA and Cy5-conjugated streptavidin. Lectins binding were evaluated by flow citometry. Representative histograms are shown. Data are the mean (S.D.), n = 3 (A) or are representative (B) of four experiments analyzed by two-tailed unpaired Student's t-test.

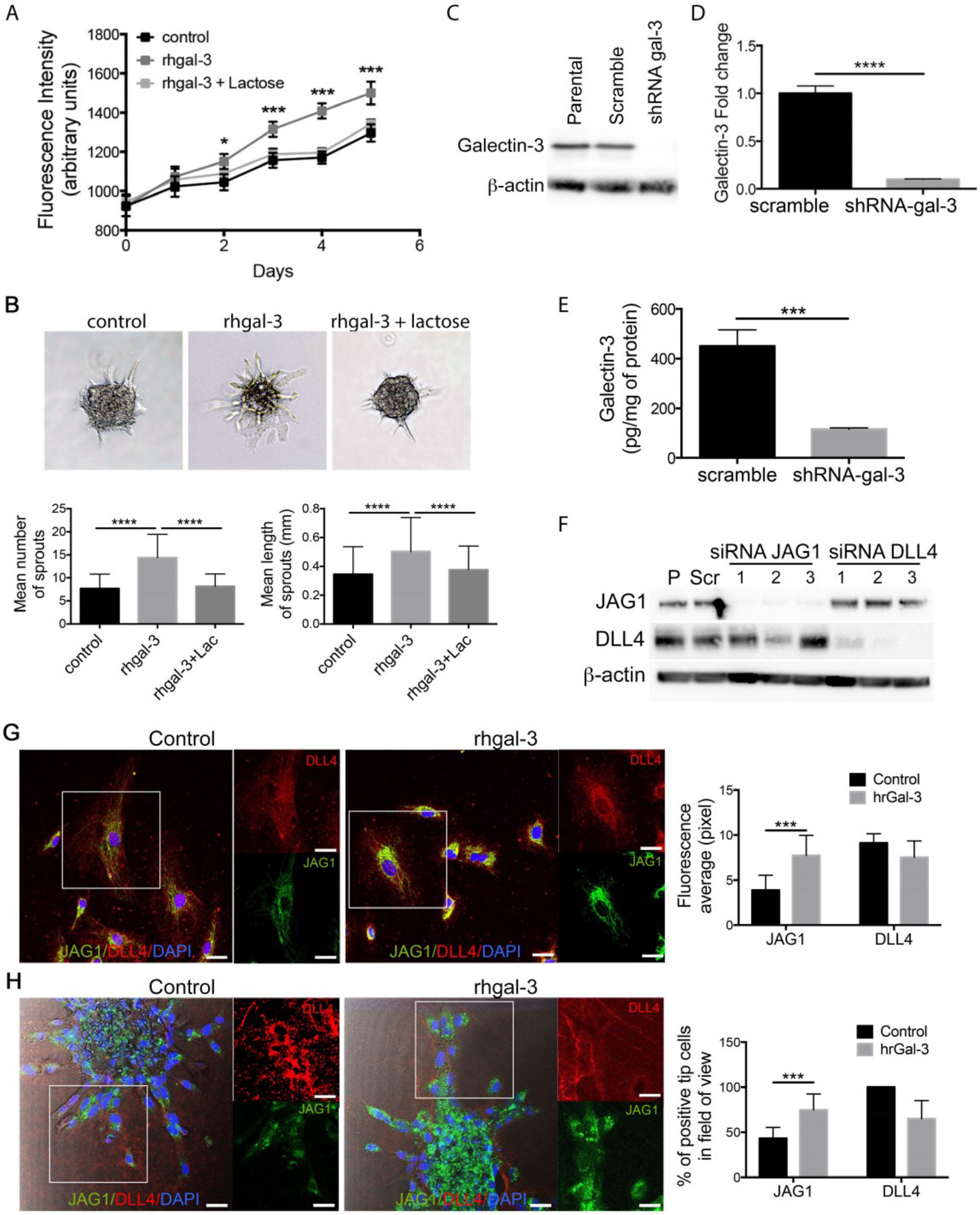

Supplementary Figure 2: Galectin-3 secretion by cancer cells increases sprouting angiogenesis via JAG1 ligand. (**A**) HUVECs were treated for 5 days with 37 nM of rhgal-3 ± lactose (50 mM). Proliferation was measured by the CyQuant assay. Data show the average fluorescent intensity of three independent experiments. (**B**) Top panel, representative figures of a sprouting angiogenesis assay with HUVECs spheroids cultured for 24 hrs in the presence of rhgal-3 (37 nM) ± lactose (50 mM). The average number and the sprouts length were quantified (bottom panel). (**C**) and (**D**) (C) Immunoblot and (D) mRNA levels of galectin-3 in MCF7 cells transduced with scramble or shRNA-gal-3 lentiviral particles. (**E**) MCF7-scramble or shRNA-gal-3 cells were cultured in a six well plate for 48 hrs. After this period, the conditioned medium of cells was collected and gal-3 from the medium was quantified by an ELISA assay. Data are presented as pg/mg of total protein. (**F**) HUVECs were treated with JAG1 or DLL4 siRNA inhibition in HUVECs for 48 hrs. After this period, total protein was isolated and JAG1 and DLL4 protein levels were evaluated by Western Blot. β-actin was used as a loading control. (**G**) HUVECs (monolayer) were treated with rhgal-3 for 6 hrs and then fixed in 4% PFA. Representative immunofluorescence images showing JAG1 (green), DLL4 (red), and nucleus (blue) staining are presented. The fluorescence average (pixel) of JAG1 or DLL4 expression was calculated using ImageJ (graph). Scale bar, 50 µm. (**H**) HUVECs spheroids were pretreated with 37 nM of rhgal-3 prior to embedding in fibrinogen gel. After 24 hrs of culture, spheroids were fixed in 4% PFA and stained for JAG1 and DLL4. Representative immunofluorescence images showing JAG1 (green), DLL4 (red), and nucleus (blue) staining are shown. The percentage number of JAG1 or DLL4 positive tip cells was evaluated (graph). Scale bar, 50 µm. Data are the mean (S.D.), (A, B, D, E, G and H), $n = 3$, or are (B, C, G and H) representative of three independent experiments. *$p < 0.05$, ***$p < 0.001$, ****$p < 0.0001$ by one-way ANOVA.

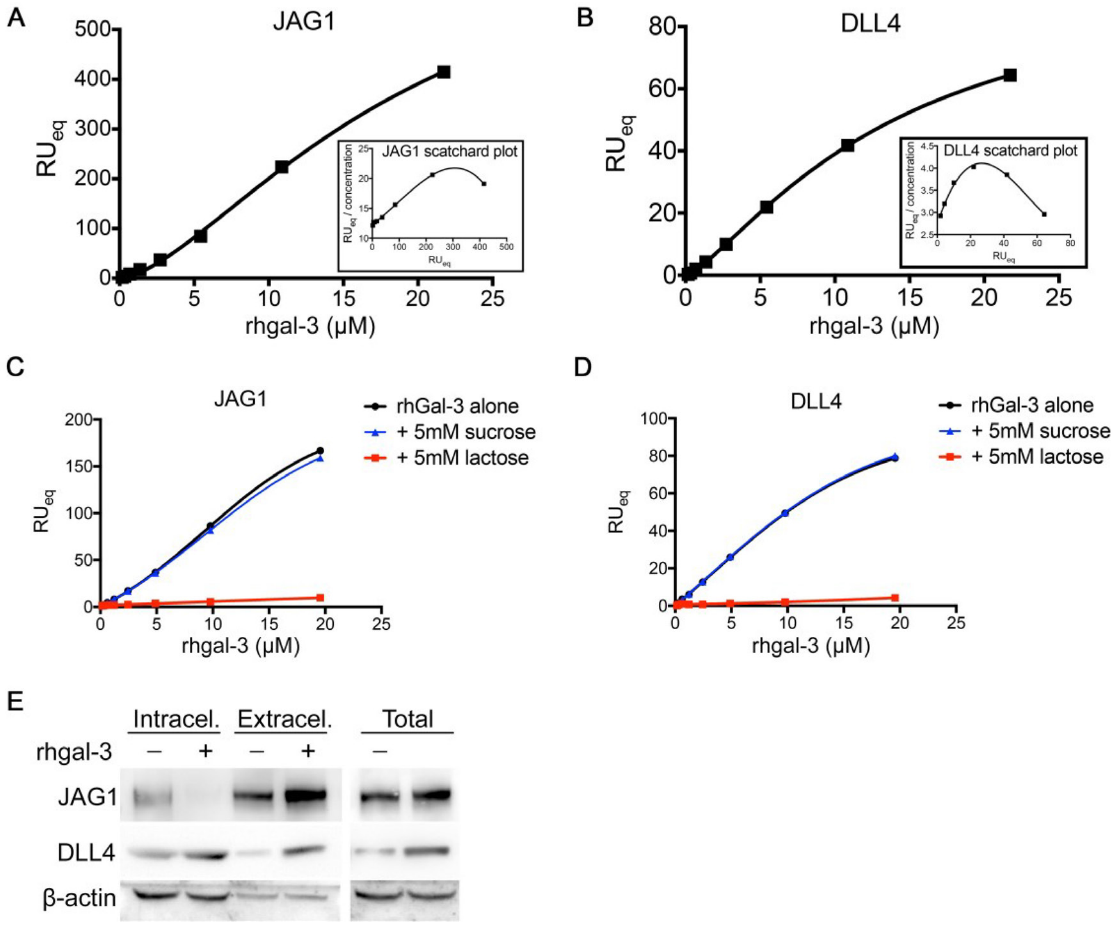

Supplementary Figure 3: Galectin-3 binds and increases JAG1 half-life over DLL4. (**A**) and (**B**) Plot of SPR binding at equilibrium against analyte concentration (with Scatchard plot inset) for a serial dilution of rhgal-3 (0.17 μM–21,7 μM) over immobilized (A) JAG1 or (B) DLL4. (**C** and **D**) SPR binding at equilibrium of rhgal-3 (0.15 μM–19.5 μM) to immobilized (C) JAG1 or (D) DLL4 in the presence of sucrose (5 mM) or lactose (5 mM). (**E**) Immunoblot of JAG1 and DLL4 after isolating the cell surface and intracellular proteins of HUVECs treated for 6 hrs with rhgal-3 (37 nM). β-actin was used as a loading control. Data are representative of four (A–D) and three (E) independent experiments.

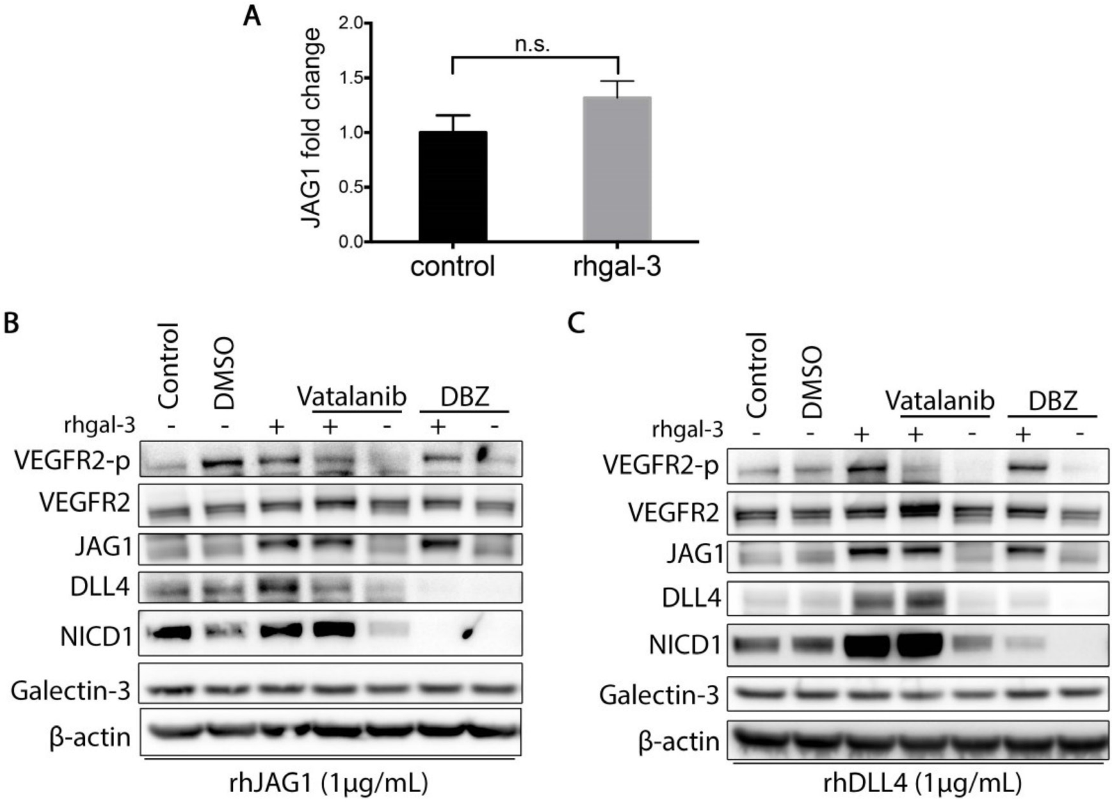

Supplementary Figure 4: Galectin-3 increases Notch signaling activation in endothelial cells. (**A**) HUVECs were cultured for 6 hrs in the presence of 37 nM of rhgal-3. After this period, total RNA was extracted and *JAG1* mRNA levels were evaluated by Real-Time PCR. Relative quantification was done using the ΔΔCt method normalizing to GAPDH gene expression. (**B** and **C**) HUVECs were cultured in the presence of Vatalanib (100 nM) or DBZ (10 nM) for 1h and then treated for 15 min with rhgal-3 (37 nM). After this period, cells were cultured for additional 6 hrs in the presence of immobilized rhJAG1 or rhDLL4. Total protein was isolated and the protein levels of galectin-3, NICD1, DLL4, JAG1, VEGFR2 and VEGFR2-p were assessed by Western blot. β-actin was used as a loading control. Data are the mean (S.D.), n = 3 and analyzed by two-tailed unpaired Student's t-test (A) or are representative of three different experiments (B and C).

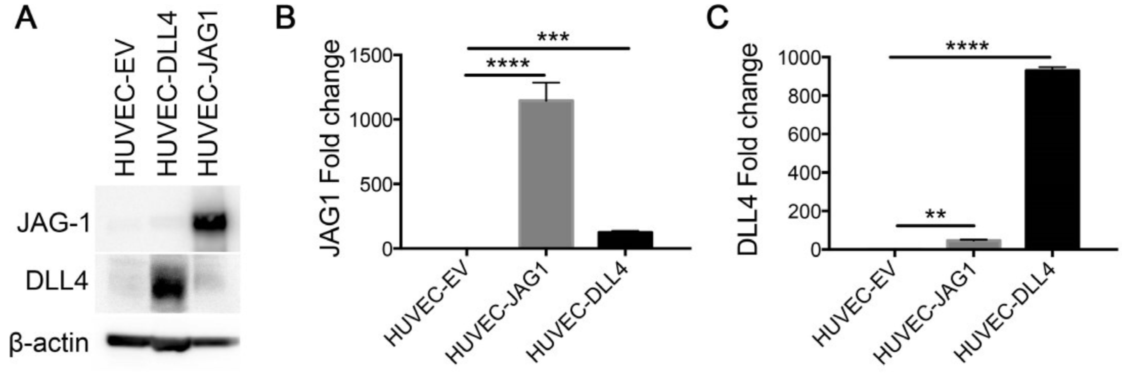

Supplementary Figure 5: Tumor-secreted galectin-3 increases JAG1/Notch signaling in endothelial cells. (**A**) Immunoblot of JAG1 and DLL4 in JAG1- or DLL4-overexpressing HUVECs. β-actin was used as a loading control. (**B** and **C**) mRNA relative expression of (B) JAG1 and (C) DLL4 in JAG1- or DLL4-overexpressing HUVECs. The relative level of expression was normalized to the level of *GAPDH* mRNA expression. Data are representative of three different experiments (A) or are the mean ± S.D., n = 3 and analyzed by two-tailed unpaired Student's t-test. $^{**}p < 0.01$, $^{***}p < 0.001$, $^{****}p < 0.0001$.

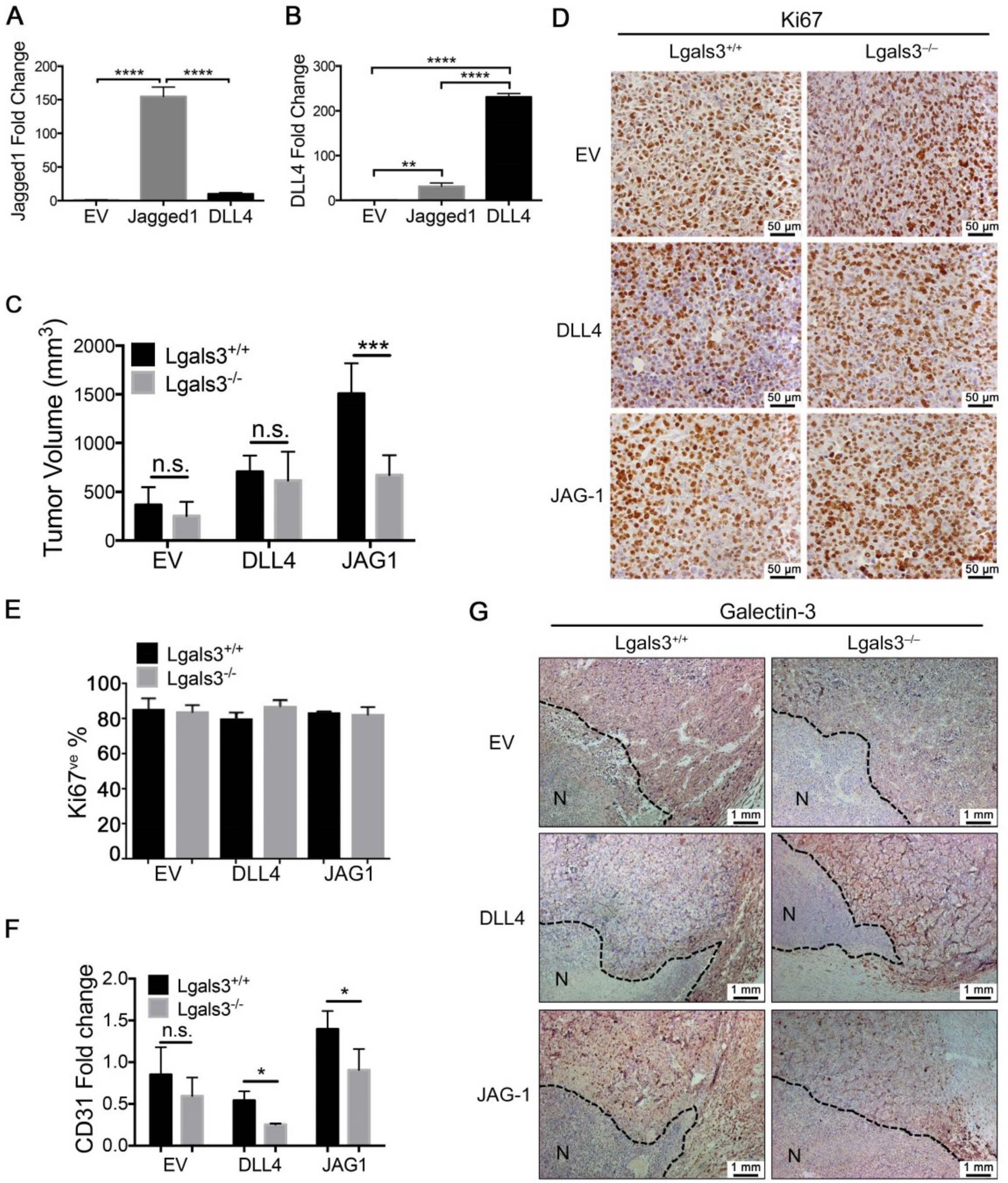

Supplementary Figure 6: The growth of JAG1 overexpressing tumor cells is impaired in galectin-3 knock out mice. (**A** and **B**). mRNA levels of (A) JAG1 and (B) DLL4 of LLC cells overexpressing the full length of murine JAG1 or DLL4. (**C**) Tumor volume of LLC-EV, LLC-JAG1 and LLC-DLL4 inoculated in C57black/6 Lgals3$^{+/+}$ or Lgals3$^{-/-}$ mice at day 18. (**D**) and (**E**) (D) Immunohistochemical staining and (E) quantification of Ki67 in LLC-EV, LLC-JG1 or LLC-DLL4 tumors inoculated in C57black/6 Lgals3$^{+/+}$ or Lgals3$^{-/-}$ mice. (**F**) CD31 mRNA levels in tumors of LLC-EV, LLC-JG1 or LLC-DLL4 derived tumors. (**G**) Immunohistochemical staining of galectin-3 in LLC-EV, LLC-JG1 or LLC-DLL4 tumors inoculated in C57black/6 Lgals3$^{+/+}$ or Lgals3$^{-/-}$ mice. Data are (A–C, E and F) the mean ± S.D., $n = 3$ or are (D and G) representative microphotograph of immunohistochemical staining. Data analyzed by one-way ANOVA or by two-tailed unpaired Student's *t*-test. *$p > 0.05$, **$p < 0.01$, ***$p < 0.001$, ****$p < 0.0001$.
